# The *Rogdi* knockout mouse is a model for Kohlschütter–Tönz syndrome

**DOI:** 10.1038/s41598-023-50870-2

**Published:** 2024-01-03

**Authors:** Alexandra Jimenez-Armijo, Supawich Morkmued, José Tomás Ahumada, Naji Kharouf, Yvan de Feraudy, Gergo Gogl, Fabrice Riet, Karen Niederreither, Jocelyn Laporte, Marie Christine Birling, Mohammed Selloum, Yann Herault, Magali Hernandez, Agnès Bloch-Zupan

**Affiliations:** 1 Institut de Génétique et de Biologie Moléculaire et Cellulaire (IGBMC), INSERM U1258, CNRS- UMR7104, Université de Strasbourg, Illkirch, France; 2 Pediatrics Division, Department of Preventive Dentistry, Faculty of Dentistry, Khon Kaen University, Khon Kaen, Thailand; 3 Laboratoire de Biomatériaux et Bioingénierie, Inserm UMR_S 1121, Université de Strasbourg, Strasbourg, France; 4 Department of Neuropediatrics, Strasbourg University Hospital, Strasbourg, France; 5 CNRS, INSERM, CELPHEDIA, PHENOMIN, Institut Clinique de la Souris (ICS), Université de Strasbourg, Illkirch, France; 6 Centre Hospitalier Régional Universitaire de Nancy, Competence Center for Rare Oral and Dental Diseases, Université de Lorraine, Nancy, France; 7 Faculté de Chirurgie Dentaire, Université de Strasbourg, Strasbourg, France; 8 Institut d’études Avancées (USIAS), Université de Strasbourg, Strasbourg, France; 9 Pôle de Médecine et Chirurgie Bucco-Dentaires, Hôpital Civil, Centre de Référence des Maladies Rares Orales et Dentaires, O-Rares, Filière Santé Maladies Rares TETE COU, European Reference Network ERN CRANIO, Hôpitaux Universitaires de Strasbourg (HUS), Strasbourg, France; 10 Eastman Dental Institute, University College London, London, UK

**Keywords:** Disease model, Translational research, Developmental biology, Genetics, Diseases, Dental diseases, Neurological disorders, Oral diseases

## Abstract

Kohlschütter–Tönz syndrome (KTS) is a rare autosomal recessive disorder characterized by severe intellectual disability, early-onset epileptic seizures, and amelogenesis imperfecta. Here, we present a novel *Rogdi* mutant mouse deleting exons 6–11- a mutation found in KTS patients disabling *ROGDI* function. This *Rogdi*^*−/−*^ mutant model recapitulates most KTS symptoms. Mutants displayed pentylenetetrazol-induced seizures, confirming epilepsy susceptibility. Spontaneous locomotion and circadian activity tests demonstrate *Rogdi* mutant hyperactivity mirroring patient spasticity. Object recognition impairment indicates memory deficits. *Rogdi*^*−/−*^ mutant enamel was markedly less mature. Scanning electron microscopy confirmed its hypomineralized/hypomature crystallization, as well as its low mineral content. Transcriptomic RNA sequencing of postnatal day 5 lower incisors showed downregulated enamel matrix proteins *Enam, Amelx,* and *Ambn*. Enamel crystallization appears highly pH-dependent, cycling between an acidic and neutral pH during enamel maturation. *Rogdi*^*−/−*^ teeth exhibit no signs of cyclic dental acidification. Additionally, expression changes in *Wdr72*, *Slc9a3r2*, and *Atp6v0c* were identified as potential contributors to these tooth acidification abnormalities. These proteins interact through the acidifying V-ATPase complex. Here, we present the *Rogdi*^*−/−*^ mutant as a novel model to partially decipher KTS pathophysiology. *Rogdi*^*−/−*^ mutant defects in acidification might explain the unusual combination of enamel and rare neurological disease symptoms.

## Introduction

Kohlschütter–Tönz syndrome (KTS, OMIM 226,750, ORPHA: 1946) is a rare autosomal recessive disorder. It is caused by mutations in the *ROGDI* gene, encoding a protein of unknown function highly conserved among diverse species, including *Caenorhabditis elegans, Drosophila melanogaster, Danio rerio, Xenopus laevis, and Mus musculus*^1–4^. First described in 1974^5^, several additional affected individuals with splice-site, nonsense, and frameshift mutations in *ROGDI* have been reported^1,2,6–8^.

KTS patients consistently display debilitating neurological deficits, including early childhood-onset epilepsy, spasticity, intellectual disability, and psychomotor regression. A dysplastic brown to yellow enamel defect marking amelogenesis imperfecta (AI) is also always seen^2,6^. Nephrocalcinosis has also been described recently as a new clinical feature in patients with KTS^9^.

Epilepsy often manifests within the first year, with seizures frequently resistant to antiepileptic treatments. While monotherapy with perampanel has provided seizure control in some patients^9^, the most severely affected individuals have profound intellectual disability, never acquire speech, and become bedridden early in life^1,2,10^. Clinical diagnosis of KTS is usually not made using neurological deficits (as these are found in numerous disorders). Hence, enamel discoloration is typically a first indicator of a KTS diagnosis^6,11^.

Amelogenesis imperfecta (AI) is a heterogeneous group of diseases affecting enamel formation^12^. While AI can present as an isolated disease, it can also coexist with other abnormalities, as observed in KTS^13^. AI is classified into four main categories (hypoplastic, hypomature, hypocalcified, hypomature hypoplastic with taurodontism) according to clinical defects and inheritance mode^14^. In *ROGDI*-associated KTS, AI is of the hypomineralization type (less mineralized, porous, rough, and brown stained in both primary and permanent teeth) with maturation defects and associated rough colored dental surfaces^11^. Disturbances during the maturation stage of amelogenesis result in pathologically softer-hypomature enamel of normal thickness. Enamel defects result in frequent caries and sensitive and esthetically disfigured teeth.

*ROGDI* is expressed in the human brain, spinal cord, blood, heart, and bone marrow^1^. Presynaptic *ROGDI* localization in rat hippocampus suggests functions in protein exocytosis^13^. ROGDI is also localized to the nuclear envelope of cultured human cells^1,8^. *Rogdi* is expressed early in the E14.5 cap stage tooth in mouse^15^, suggesting developmental roles. Structural analysis further reveals truncated Rogdi proteins present in KTS patient^2,6^ are likely unstable and likely degraded^3^.

Here we report a mouse mutant model of *Rogdi* inactivation recapitulating typical KTS patient variants^12^. This *Rogdi*^*−/−*^ mutation recapitulates most KTS patient symptoms—epilepsy, hyperactivity, memory deficits, and markedly less mature hypomineralized/hypomature enamel of low mineral content.

## Results

### *Rogdi* expression analysis

**Figure 1:**
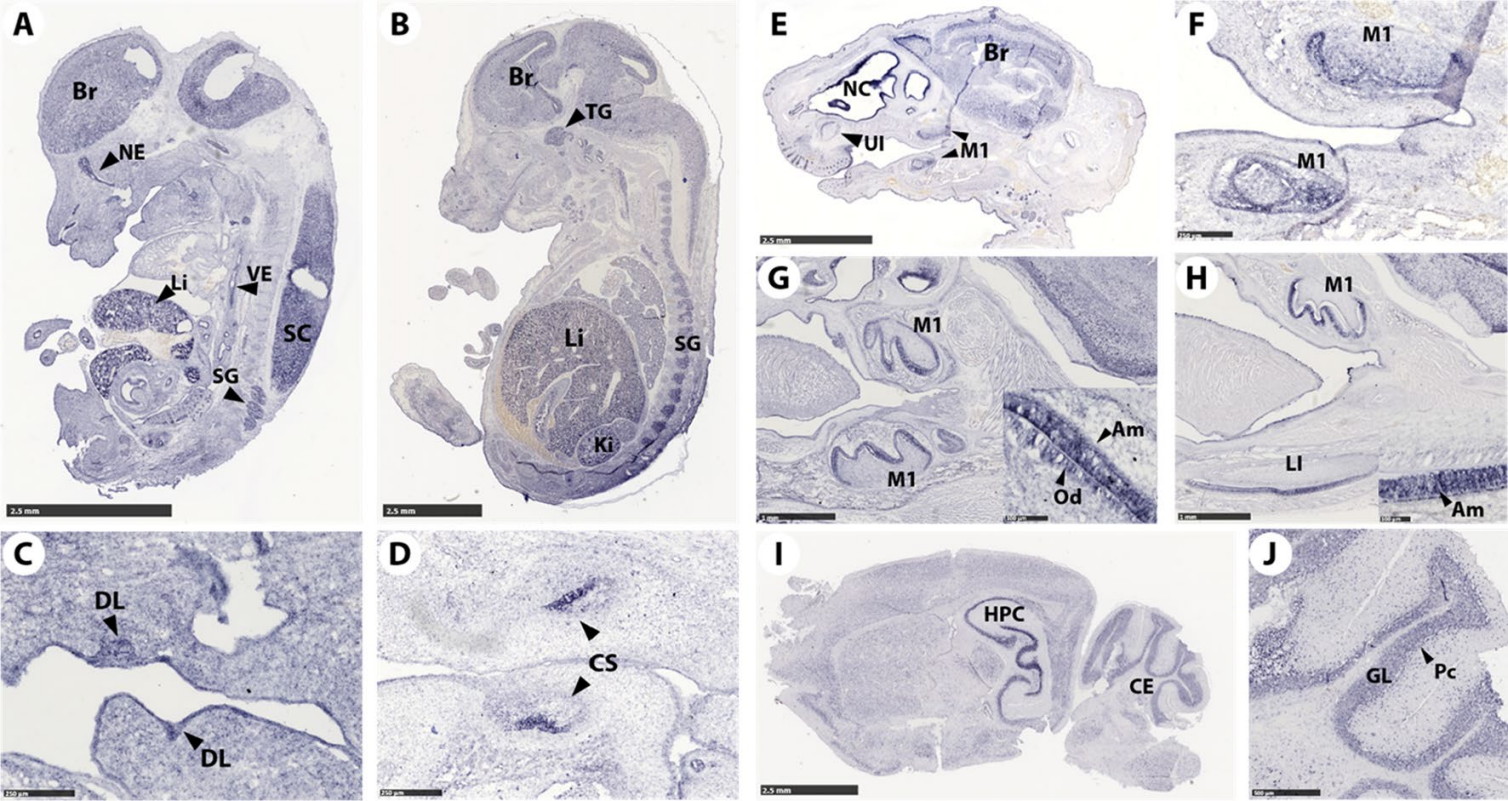
*Rogdi* expression. (**A**) *Rogdi* mRNA is expressed in the brain (Br), nasal epithelium (NE), spinal cord (SC), spinal ganglion (SG), and liver (Li) at E12.5. (**B**) At E14.5, continued enriched expression in the brain and liver is observed, and trigeminal ganglion (TG), spinal ganglion (SG), and kidney (Ki) *Rogdi* enrichment is observed. *Rogdi* odontogenic expression begins at (**C**) E12.5 in the dental lamina (DL), (**D**) continues in the E14.5 cap stage (CS) and in (**F**) E16.5 bell stage molars. (**E**) Expression of *Rogdi* mRNA at E16.5 in the brain, nasal cavity (NC), vibrissae (VB), upper incisor (UI) and first molar (M1) at the bell stage. (**G**) At postnatal day P1, *Rogdi* mRNA is present in ameloblasts and odontoblasts of the lower molar. (**H**) At P5, enriched expression in ameloblasts appears (compare insert panels in G [P1] vs. H [P5] showing P5 ameloblast enrichment. (**I**) Adult mouse brain. Pronounced expression of Rogdi in the hippocampus (HPC) and in the cerebellum (CE) is observed. (**J**) In the cerebellum, *Rogdi* appears to be enriched in the granular layer (GL) and Purkinje cells (Pc).

*Rogdi* transcript location analysis in wild-type (WT) mice by in situ hybridization experiments at embryonic (E) 8.5–10.5-stage of post coitus development—indicated no early differential domains of *Rogdi* expression. Starting at E12.5, we detected enriched *Rogdi* transcripts in neuroepithelium tissues, including the brain, spinal cord, and spinal ganglion (Fig. 1A). Differential expression in the liver and vascular endothelium was also apparent at this stage. At E14.5 (Fig. 1B), central nervous system expression, including in the trigeminal and spinal ganglia, persisted. Liver and kidney enrichments were also observed (Fig. 1B).

In the 7-week-old adult brain, enriched *Rogdi* expression is detected in both the hippocampus (HPC) and cerebellum (CE) (Fig. 1I,J). Our reanalysis of single-cell transcriptomics data of central nervous system cell populations^16^ found *Rogdi* enrichment in corticostriatal neurons, corticospinal neurons, neurons (CCK), stellate and basket cells, unipolar brush cells, and granule cells (see Supplementary Table S1).

During tooth development, transcripts were localized from the dental lamina at E12.5 (Fig. 1C), in the cap stage at E14.5 (Fig. 1D), in the bell stage at E16.5 (Fig. 1E,F), and at postnatal PN1 (Fig. 1G) in both the ectodermal and ectomesenchymal compartments. *Rogdi* displayed differential enrichment in both ameloblasts and odontoblasts. At PN5 (Fig. 1H), *Rogdi* was enriched only in ameloblasts. Consistently using the https://kleintools.hms.harvard.edu/tools/springViewer_1_6_dev.html?datasets/Sharir_et_al_2019/control_epithelial dataset^17^, *Rogdi* transcripts are enriched in the incisor epithelium, specifically in the preameloblast and ameloblast classes. Here *Rogdi* transcript levels are 100 times lower than the *Ambn* and *Enam* enamel matrix proteins but are comparable to the levels of *Orai1* and *Stim1*, genes responsible for syndromic hypomature AI^18–20^.

**Figure 2:**
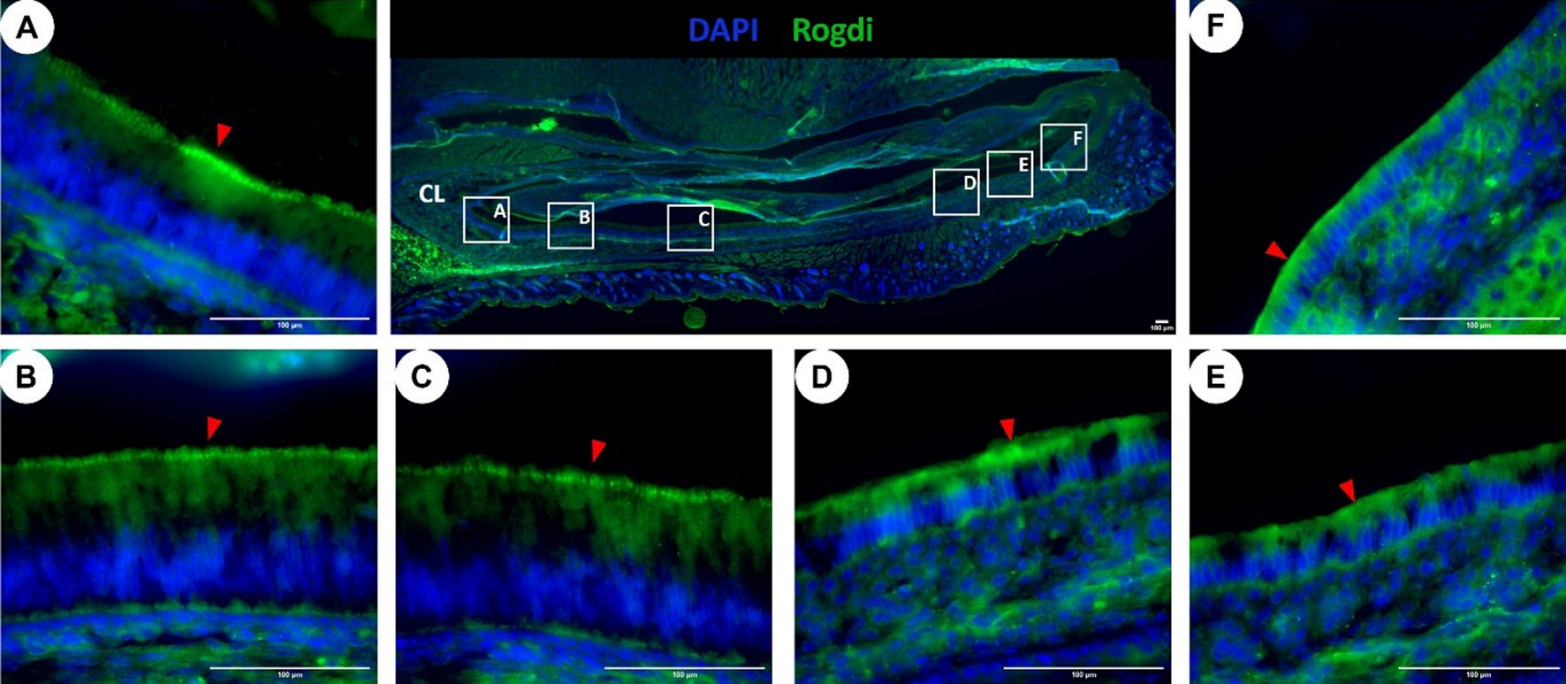
Rogdi protein localization in the postnatal 7-day lower incisor (sagittal section). Immunodetection of Rogdi in lower incisor ameloblasts showed (**A**–**C**) secretory ameloblasts with positive staining at the apical border in Tomes’ processes. (**D**–**F**) Maturation-stage ameloblasts present a more diffuse signal of Rogdi at the apical pole. Boxes A-F show the location in the lower incisor images (**A**–**F**). Red arrowheads point towards Rogdi immunolocalization at the apical pole of ameloblasts. Cervical loop (CL) appears on the left side of the image.

Immunohistochemistry shows ROGDI protein is enriched in the ameloblast Tomes’ process during the secretory stage of amelogenesis (Fig. 2A–C), and present at the apical pole of transition and maturation stage ameloblasts (Fig. 2D–F respectively).

### Creating a ***Rogdi***^*−****/−***^ mouse model

**Figure 3:**
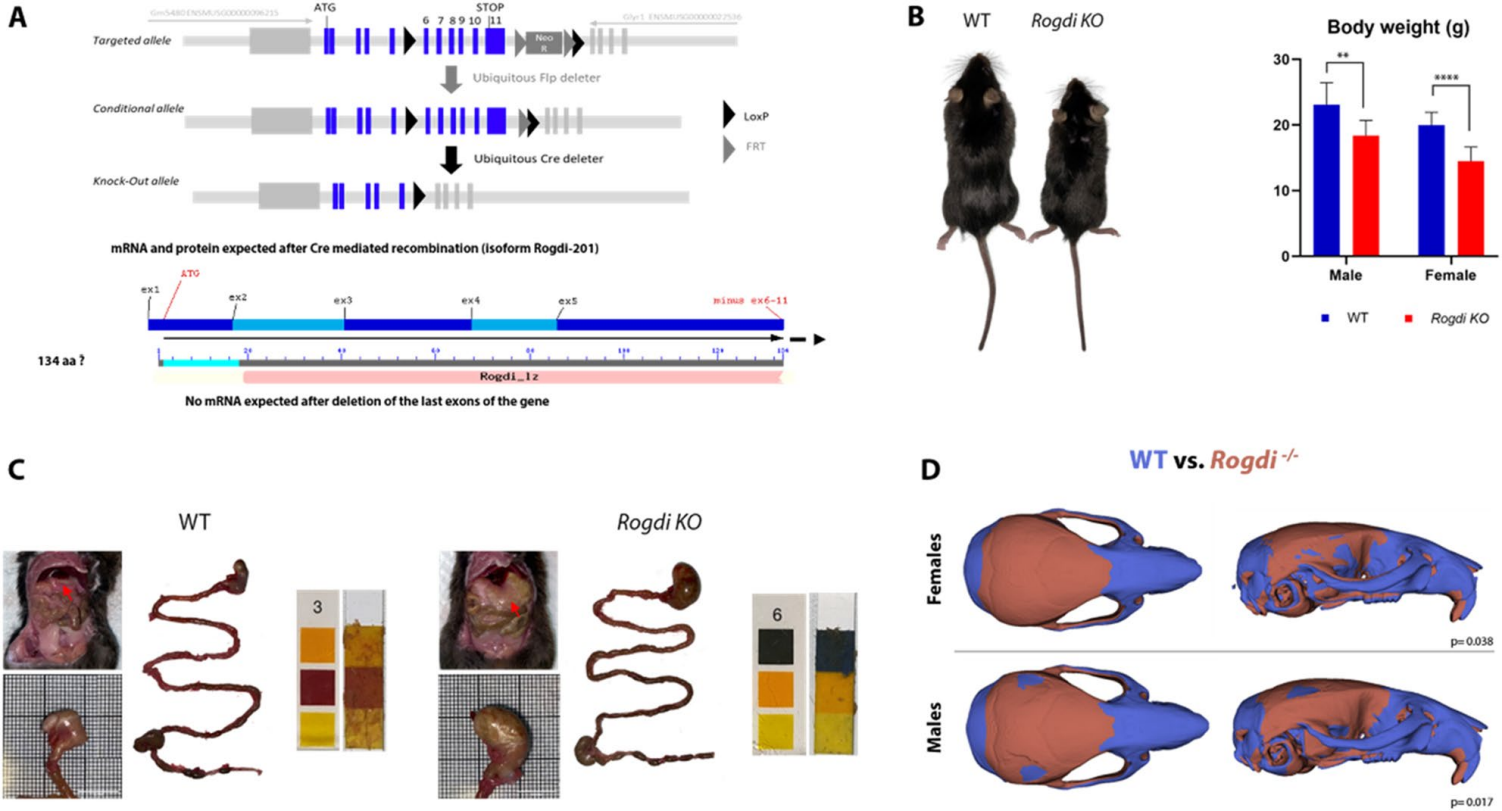
*Rogdi*^*−/−*^ mouse model. (**A**) Illustrates the mutation strategy and the protein expected after Cre-mediated recombination consisting of a truncated 134 amino acid protein. (**B**) Representative picture of an 8-week-old *Rogdi* knockout mouse (left) and a control littermate (right). The column graph on the right shows the body weights of *Rogdi* knockout female and male mice and control littermates between 8 and 12 weeks old. (**C**) Representative picture of the abdominal area of a WT and *Rogdi KO* mouse (red arrow illustrates stomach) reveals impairment in stomach emptying in *Rogdi KO* mouse. *Rogdi KO* presents an enlarged stomach compared to WT. Measurement of the pH (Merck Acilit^®^ pH Indicator Strips; 0–6 pH Range) of stomach contents indicates a near neutral pH (6) in *Rogdi KO*, whereas WT has a normal acidic pH (3). No other abnormalities were found in the gastrointestinal tract. (**D**) Form (volume + shape) differences between WT and *Rogdi *^*−/−*^. WT are displayed in blue and *Rogdi*^*−/−*^ in red. In the figure WT versus *Rogdi*^*−/−*^ rigid alignment, blue zones represent the decreased volume in *Rogdi*^*−/−*^. In red, the bones with increased volume (frontal and parietal bone) in *Rogdi*^*−/−*^. *Rogdi*^*−/−*^ males and females present a similar form. Scale bar: 10 mm. **p < 0.01 ****p < 0.0001.

To investigate the role of Rogdi in development and disease, we created a deletion model targeting exons 6 to 11 (isoform Rogdi-201, ENSMUST00000023191) by homologous recombination (see Fig. 3A for the *Rogdi* knockout construct and Supplementary Figure S1 for the genotyping strategy). The deleted exons (6–11) are at a region predicted to not escape nonsense-mediated RNA decay (NMD), so any change in the mRNA will be detected during transcription and the RNA will be destroyed rather than translated into protein. Besides, based on crystallographic analysis, the *Rogdi* null mutant would be predicted to produce a 134 amino acid nonfunctional degraded protein, disrupting the alpha domain of Rogdi, which could impair protein stability, causing loss of function^3^. This recapitulates KTS patient defect reported in Bloch-Zupan et al.^12^. While *Rogdi* mouse mutants were viable at early stages, in heterozygous crosses only around 20% were *Rogdi*^*−/−*^ (Supplementary Table S2). No gross embryonic (E9.5, E12.5) or fetal (E14.5–18.5) malformations were observed in *Rogdi*^*−/−*^ mutants. However, postnatal lethality was observed, with more than 64% of *Rogdi*^*−/−*^ mutants dying before 12 weeks of age. After weaning, the *Rogdi*^*−/−*^ mutants had significantly growth and weight reductions compared to WT (Fig. 3B). Alterations continuing throughout life. The parameters—body temperature (°C), body position, tremor, palpebral closure, coat appearance, whiskers, lacrimation, and defecation—were comparable between genotypes. The oldest living *Rogdi*^*−/−*^mouse was 49 weeks old.

To understand the early lethality of mutants, necropsy examination of 26 *Rogdi*^*−/−*^ was performed using the protocol described in Scudamore et al.^21^. This showed a distended stomach full of undigested food in 80% of the *Rogdi*^*−/−*^ mice. *Rogdi*^*−/−*^ stomach content mice pH was 6 (instead a normal acidic pH of 3). Bowel appeared normal with fecal matter present throughout (Fig. 3C compare WT and *Rogdi KO*). One *Rogdi*^*−/−*^ mouse presented hepatomegaly with an abnormal pale color in both kidneys. Another *Rogdi*^*−/−*^ had a similar renal defect. 3 *Rogdi*^*−/−*^ had a urinary bladder full of urine and with a viscous liquid inside. No other organ particularities were noted.

Micro-CT imaging (μ-CT) was performed for morphometric analysis of 8-week-old *Rogdi*^*−/−*^ mutant and WT mouse skulls. The form (size and shape) of both males and females in *Rogdi*^*−/−*^ mice was significantly different from that in WT mice. An overall reduction in head dimensions was found, with a significant decrease in dimensions in the nasal bone, premaxillary bone, maxillary bone, temporal bone (with the squamosal portion), and occipital bone. Additionally, increased volume was found in the frontal bone with an anterior projection, and in the parietal bones with a wider width (Fig. 3D), mimicking the frontal and parietal bossing of the human phenotype^2,22,23^.

Blood chemistry parameters measured on the plasma showed significantly lower levels of calcium in *Rogdi*^*−/−*^ males and higher levels of alkaline phosphatase only in *Rogdi*^*−/−*^ females. No differences between groups were found in blood cell count.

### Locomotory/grip defects, hyperactivity, memory impairment and epilepsy in *Rogdi* knockout mice

**Figure 4:**
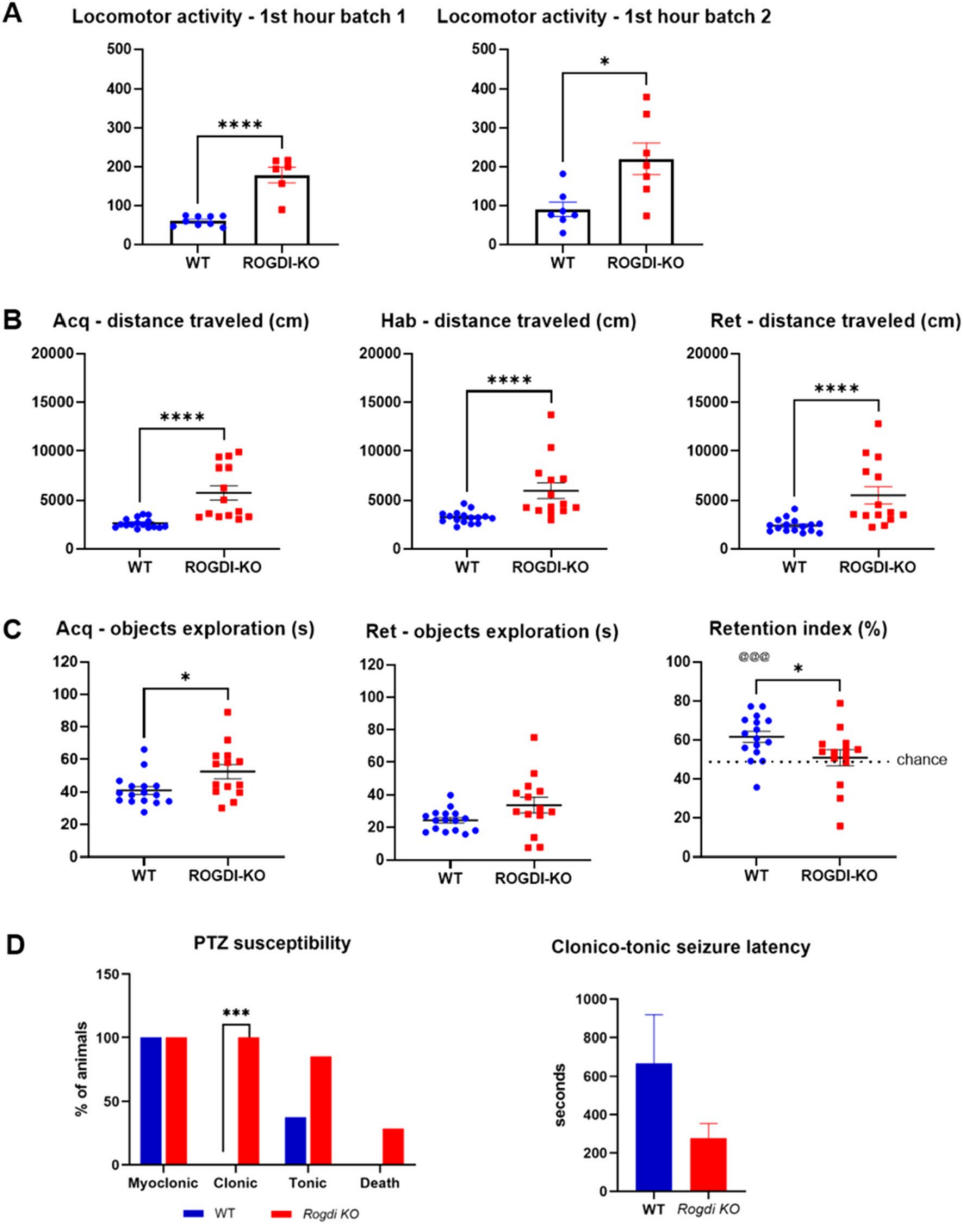
Locomotor activity, memory assessment and epilepsy susceptibility of adult WT versus *Rogdi *^*−/−*^. (**A**) Locomotor activity during the first hour of the circadian activity test. A batch effect was observed, with a higher significance in the first batch. (**B**) Locomotor activity (distance) in the whole arena during the habituation, acquisition and retention sessions of the novel object recognition test (NOR). (**C**) NOR: Exploration time (s) of the objects during the acquisition (Acq) that was significantly increased in *Rogdi *^*−/−*^ mice and retention (Ret) sessions with no difference between groups. The recognition index (%) was significantly decreased in *Rogdi *^*−/−*^ mice. This was higher than by chance for the WT mice, whereas it was not significantly different for *Rogdi *^*−/−*^ mice. (**D**) PTZ test results show that a higher percentage of *Rogdi* knockout mice developed clonic and tonic convulsive seizures compared to the control group (WT). WT did not present clonic seizures. *Rogdi KO* mice had a shorter latency for clonic-tonic seizures. Data are expressed as the mean ± SEM. *p < 0.05; ***p < 0.001, ****p < 0.0001, @@@*p* < 0.001 versus chance.

Behavioral tests evaluating motor and memory impairment in *Rogdi*^*−/−*^ and respective controls showed significant increases in locomotor activity during the first hour of the circadian activity test and during habituation (Hab), acquisition (Acq), and retention (Ret) sessions of the novel object recognition test (NOR) (Fig. 4A,B), which could be interpreted as hyperactivity disorder in the mutant mice, a feature already described in KTS^4^. Cognitive impairment assayed by NOR showed that *Rogdi*^*−/−*^ mutants exhibited memory impairment (Fig. 4C). Object exploration time was significantly increased in *Rogdi*^*−/−*^ mutants during the acquisition session, probably a consequence of hyperactivity here too. Nevertheless, a significantly decreased recognition index in *Rogdi*^*−/−*^ mutants was found with no difference from chance (Fig. 4C).

Motor function was tested using grip strength and rotarod tests. Sex and batch effects were observed in the grip strength test (Supplementary Figure S2), where in the second group of *Rogdi*^*−/−*^ females, muscle strength was significantly decreased (Supplementary Figure S2B). The rotarod test indicated no differences indicating no motor impairment between groups (Supplementary Figure S2C). The elevated plus maze (EPM) test was used to assess anxiety-related behavior and showed no differences between the genotypes (Supplementary Figure S2D). Histological analyses in the tibialis anterior (TA), gastrocnemius (Gas) and quadriceps (Qua) muscles revealed no variation in the muscle/body weight ratio between *Rogdi*^*−/−*^ and WT mice (Supplementary Figure S3A). MinFeret and small fiber percentages were similar between both groups (Supplementary Figure S3B and S3C). Histological cross sections of TA, Gas and Qua muscles from both groups showed no morphological differences (Supplementary Figure S3D). Scanning electron microscopy (SEM) of the tibialis anterior, gastrocnemius, and quadriceps muscles showed no differences between the groups.

No spontaneous seizures were detected in *Rogdi*^*−/−*^ mice. We assessed *Rogdi*^*−/−*^ susceptibility to develop induced epilepsy using a timed pentylenetetrazol infusion test (PTZ). *Rogdi*^*−/−*^ mutant animals exhibited epilepsy susceptibility and faster seizure onset. All mice showed myoclonic seizures, although only *Rogdi*^*−/−*^ mutants developed clonic seizures. At the end of the convulsion, less than 40% of the WT mice (3 of 8) presented a tonic seizure, whereas more than 90% of the *Rogdi*^*−/−*^ mutant mice (6 of 7) had tonic seizures (Fig. 4D).

### Altered cerebellar and hippocampal morphology

Cerebellar and hippocampal histology was performed at PN15. Several regional alterations in cerebellar morphometry were observed in *Rogdi*^*−/−*^ versus WT mice (Table 1). *Rogdi* mutants displayed decreased the thickness of the simplex molecular layer (Mol) (*p* < 0.01) and crus II internal granular layer (IGL) (*p* < 0.0001), while the external granular layer (EGL) of simplex (*p* < 0.05) and paramedian (*p* < 0.001) lobules was thicker than that of WT. No alteration was observed in the vermal lobule IV/V and crus I lobule.

**Table 1:** Thickness variations for granular and molecular layers of cerebellar cortex lobules in PN15 WT versus *Rogdi*^−/−^ mice.

Cerebellum					
	Vermal lobule IV/V	Simplex	Crus I	Crus II	Paramedian
IGL	− 11.9%	− 4.3%	2.5%	− 23.4%****	0.8%
Mol	− 1.7%	− 6.8%**	−1.9%	21.2%*	3.4%
EGL	3.6%	23.1%*	30% ^#^	21.2%*	69%***

The Purkinje cell layer is included in the molecular layer measure. IGL, Internal granular layer; Mol, Molecular layer; EGL, External granular layer.

^#^*p* = 0.05; *p < 0.05; **p < 0.01; ***p < 0.001; ****p < 0.0001 (unpaired Student’s t Test).

In the hippocampus, the thickness of the Oriens layer of the CA1 region was decreased in *Rogdi*^*−/−*^ mice (*p* < 0.001), while the granular layer of the dentate gyrus was thicker (*p* < 0.01) compared to control mice (Table 2).

**Table 2:** Percentage of thickness variation measured in subregions of the hippocampal formation of PN15 *Rogdi*^*−/−*^ mice relative to wild-type mice.

Hippocampal formation				
CA1			Dentate gyrus	
Oriens	Pyramidal	Molecular and radiatum	Granular	Molecular
− 24.1%***	6.1%	− 3.4%	25.7%**	2.4%

**p < 0.01; ***p < 0.001 (unpaired Student’s t Test).

### Amelogenesis Imperfecta-like dental defects

**Figure 5:**
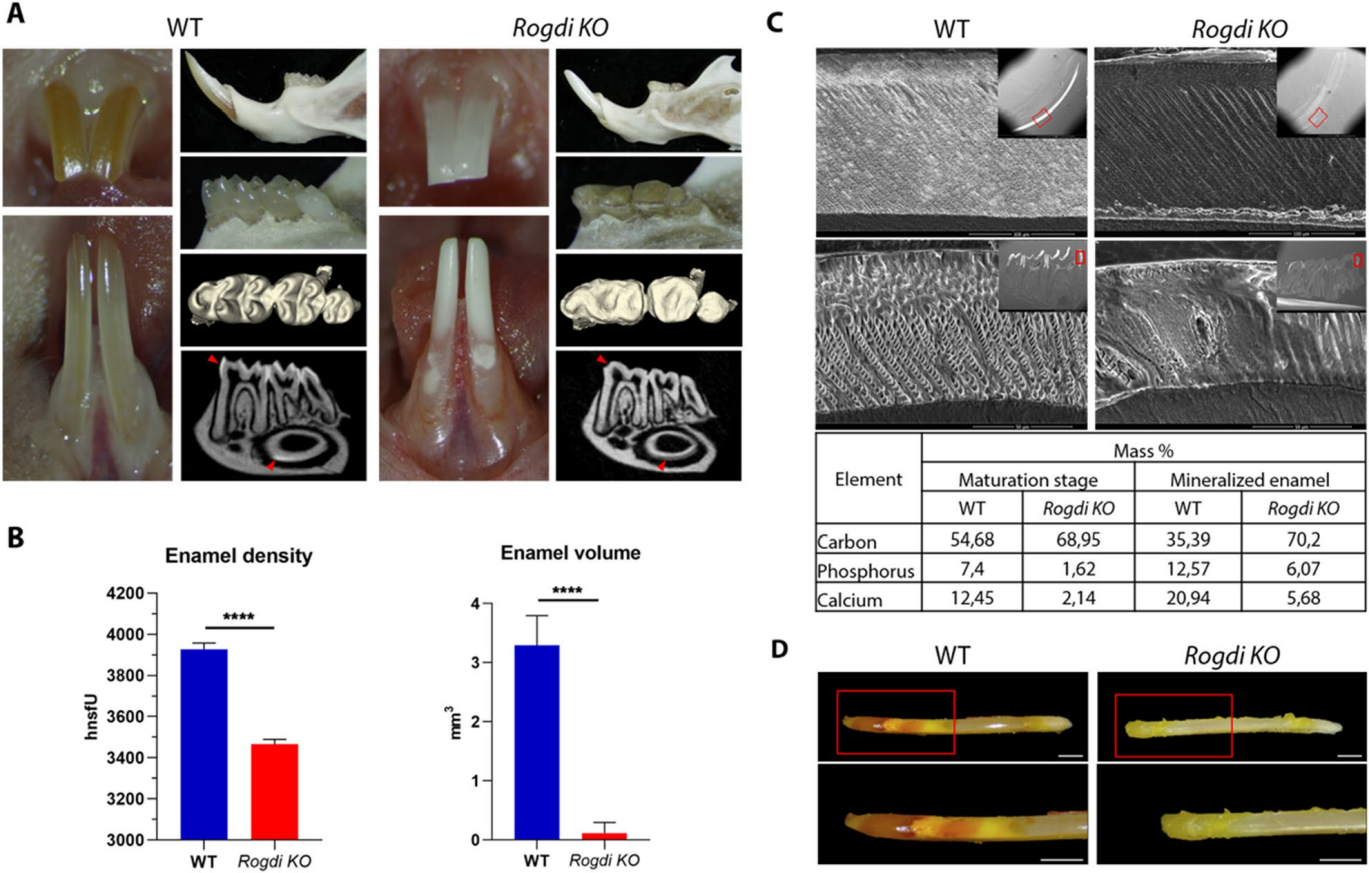
*Rogdi* tooth phenotype. (**A**) Comparison of incisors of WT 8-week-old mice, with darker yellow/orange pigmentation in the upper incisor, to incisors of 8-week-old *Rogdi*^*−/−*^ mutant mice, which have a chalky white color. *Rogdi*^*−/−*^ mutant lower incisors show chalky lightening with white patches in the cervical tooth zone. The *Rogdi KO* mouse presented abraded cusps in the molars that were severely worn, losing enamel at occlusal surfaces and exposing the dentin that remained relatively intact, confirming the enamel phenotype. Optical sections in a sagittal plane show reduced enamel mineral density in the lower molars and incisor (red arrowheads). (**B**) Analysis of enamel density (HnsfU) and volume (mm^3^) shows that *Rogdi KO* enamel is highly diminished and less dense than that of WT mice. (**C**) Scanning electron microscopy (SEM) imaging of 8-week-old WT and *Rogdi*^*−/−*^ teeth shows a reduced mineral density of enamel in both incisors and molars in *Rogdi* KO. Red boxed regions in insert panels show regions in which higher magnification of SEM images were obtained. The enamel of *WT* presents a constant thickness and a clear decussating prism pattern, while the *Rogdi*^*−/−*^ variant produces a near complete absence of opaque mineralized enamel matrix. Table of energy dispersive X-ray spectrometry (EDX) data for quantification of element composition of enamel in maturation stage of amelogenesis and mineralized enamel of lower incisor. The calcium and phosphate concentrations in the enamel layer of the wild-type mouse are normal, but both concentrations are highly diminished in the *Rogdi* mutant. Carbon levels are higher in *Rogdi* KO, suggesting a lack of enamel matrix protein degradation during the maturation stage of amelogenesis. (**D**) Methyl-red staining in 12-week-old lower incisors. In wild-type mice, secretory-stage enamel stained neutral (yellow) with methyl-red. In maturation-stage enamel, methyl-red staining revealed 2 broad acidic bands in control mice, the first intensely deep-red and the second (incisal) weaker. No red bands (acidic) were noted in the enamel of *Rogdi KO* mice. ****p < 0.0001. Scale bar: 1 mm.

At adult stages, *Rogdi*^*−/−*^ mutants had clear macroscopically visible AI-like tooth defects. The labial surface of the rodent incisor (the crown analog covered by enamel) is normally yellow/orange due to 0.03% enamel iron content^24^. This characteristic color dark orange upper incisors enamel pigmentation, is in contrast chalky white in *Rogdi*^*−/−*^ mutant incisors (Fig. 5A, compare WT vs. *Rogdi*^*−/−*^). *Rogdi*^*−/−*^ mutants (compared to WT, Fig. 5A) white patches showed fragmentation or "chipping" more pronounced at the bases of the lower incisors. Molars were abraded with enamel loss on the occlusal side exposing the dentin, although the animals were on a soft diet.

µCT imaging showed an overall reduction in optical density (darker appearance of mutant enamel), revealing reduced enamel mineral density in both incisors and molars (Fig. 5B, Supplementary Figure S4 and S5). *Rogdi* mutation produced a near complete absence of opaque mineralized enamel matrix.

To assay overall structural changes, *Rogdi*^*−/−*^ mutant samples were analyzed using scanning electron microscopy (SEM) imaging. Enamel thickness in both the upper incisors and lower molars was normal in *Rogdi*^*−/−*^ mutants (Fig. 5C), suggesting that EMP secretion was intact. The prismatic organization and the intermingled decussation pattern were conserved in *Rogdi*^*−/−*^ enamel (compare WT and *Rogdi*^*−/−*^ in Fig. 5C). Enamel of wild-type incisors showed normal crystallization, whereas *Rogdi* null incisors were undermineralized (Fig. 5C). X-ray energy dispersion spectrometry (to determine overall mineral content) indicated that the enamel matrix indeed had a reduced mineral content in *Rogdi*^*−/−*^ mutants. Calcium levels in maturation stage and fully mineralized stage enamel were reduced by more than 80% and 70%, respectively, compared to controls. Phosphorus levels were also diminished at both stages, with approximately 80% in maturation and 50% in final mineralized enamel. Carbon levels, indicative of organic content, were higher in mutants and did not differ between maturation stage and mineralized enamel in *Rogdi*^*−/−*^ mutants (Table in Fig. 5C), suggesting a lack of enamel matrix degradation during the maturation stage of amelogenesis. SEM analysis was also performed in teeth from one of the patients with KTZ from the Reference Center for Rare Orodental Diseases, CRMR O-Rares. Canine (23) did not present enamel. Enamel in premolar (45) was present but hypoplastic. The calcium-phosphate ratio was 1.65 (Supplementary Figure S6), while normal values are approximately 2.17 ± 0.1^25^. These data showed that the mineral content in KTS teeth is diminished, which is consistent with the observed phenotype in *Rogdi* null mice.

### Defective incisor acidification in *Rogdi* mutants

pH modulation is extremely important during enamel formation, especially at the maturation stage where ameloblasts modulate and alternate between a smooth end and a ruffle border, involving repetitive pH changes in the forming enamel seen as acid (pH 6.0) and neutral (pH 7.2) bands. To explore these steps, methyl red staining was performed in 12-week-old lower incisors for pH analysis. Methyl red stains red at acidic pH (~ 5.0–5.8) and yellow at neutral pH (~ 6.6–8.6). The results revealed a clear red acidic band at the maturation stage in WT mice (Fig. 5D). However, in *Rogdi*^*−/−*^ mutant enamel, no acid bands appeared, staining all the enamel yellow (Fig. 5D), showing no changes in pH during amelogenesis.

### RNA sequencing analysis of postnatal 5 (PN5) incisors

To track global gene expression alterations, independent lower incisors of PN5 male *Rogdi* mutants and WT controls were analyzed by high-throughput RNA sequencing (RNA-seq). Principal component analysis (PCA plot, Supplementary Figure S7A) revealed a clear separation between *WT* and *Rogdi*^*−/−*^ mutant samples. Bioinformatic analysis was performed by selecting significant genes using a *p*-value < 0.05 and an absolute value of log2 fold-change > 0.3, providing volcano plots (Supplementary Figure S7B) plotting log2 fold-change on the X-axis versus statistical significance. RNA-seq data confirmed the downregulation of *Rogdi* transcripts, affecting neurodegeneration and synapse pathways, calcium signaling, tight junctions, Wnt, Notch and Hedgehog signaling networks, among others (Supplementary Figure S7C).

The enamel matrix proteins (EMPs), amelogenin (AMEL), ameloblastin (AMBN), and enamelin (ENAM)—regulatory targets are reduced in *Rogdi* mutants (Table 3).

**Table 3:** RNA-sequencing data.

Gene name	Description	Log2 FC (*Rogdi*^*−/−*^ vs. WT)	*p*-value
Amelogenesis			
*Lama3*	laminin. alpha 3	− 1.01	8.8E−12
*Itgb6*	integrin beta 6	− 0.89	5.1E−09
*Enam*	enamelin	− 0.66	1.0E−04
*Acp4*	acid phosphatase 4	− 0.65	1.9E−04
*Amelx*	amelogenin. X-linked	− 0.63	4.2E−04
*Ambn*	ameloblastin	− 0.62	2.9E−04
*Lamb3*	laminin. beta 3	− 0.60	1.4E−05
*Shh*	sonic hedgehog	− 0.37	3.2E−02
*Col17a1*	collagen. type XVII. alpha 1	− 0.36	7.3E−06
*Itgb4*	integrin beta 4	− 0.33	8.0E−04
*Car12*	carbonic anhydrase 12	0.30	3.3E−02
*Cnnm4*	cyclin M4	0.30	4.3E−03
*Amtn*	amelotin	0.35	2.4E−02
*Ltbp3*	latent transforming growth factor beta binding protein 3	0.37	3.6E−04
*Slc24a4*	solute carrier family 24 (sodium/potassium/calcium exchanger). member 4	0.38	2.8E−02
*Lamb2*	laminin. beta 2	0.39	5.5E−04
*Wdr72*	WD repeat domain 72	0.40	2.6E−02
*Gpr68*	G protein-coupled receptor 68	0.44	6.6E−05
*Orai1*	ORAI calcium release-activated calcium modulator 1	0.47	4.6E−05

Data are presented as log2-fold changes in *Rogdi*^*−/−*^ versus WT samples. For instance, a FC log2 value of − 1.00 will correspond to a 50% reduction in mRNA level in the *Rogdi*^*−/−*^ samples. Genes encoding regulators of amelogenesis are either reduced or increased in mutant samples.

Downregulated amelogenesis imperfecta-implicated genes at PN5 included *Lama3, Itgb6, Acp4, Lamb3* and *Col17a1*. Other amelogenesis genes, such as *Car12, Cnnm4, Amtn, Ltbp3, Slc24a4, Lamb2, Gpr68* and *Orai1,* were upregulated. V-ATPase complex genes *Slc9a3r2* and *Atp6v0c*^26,27^ and *Wdr72* (a homologue of WDR7^28^) were also upregulated (Table 3 and Supplementary Table S3).

*Rogdi* transcripts were also analyzed by qPCR, showing a 4.5-fold lower expression fold change of exons 1–4 in the *Rogdi*^*−/−*^ group than in the WT group, and a 1500-fold lower expression fold change of exons 6–11 in the *Rogdi*^*−/−*^ group (Supplementary Figure S7D). RNA-seq data showed an eightfold reduction (log2 FC-2.97) of *Rogdi*.

**Figure 6:**
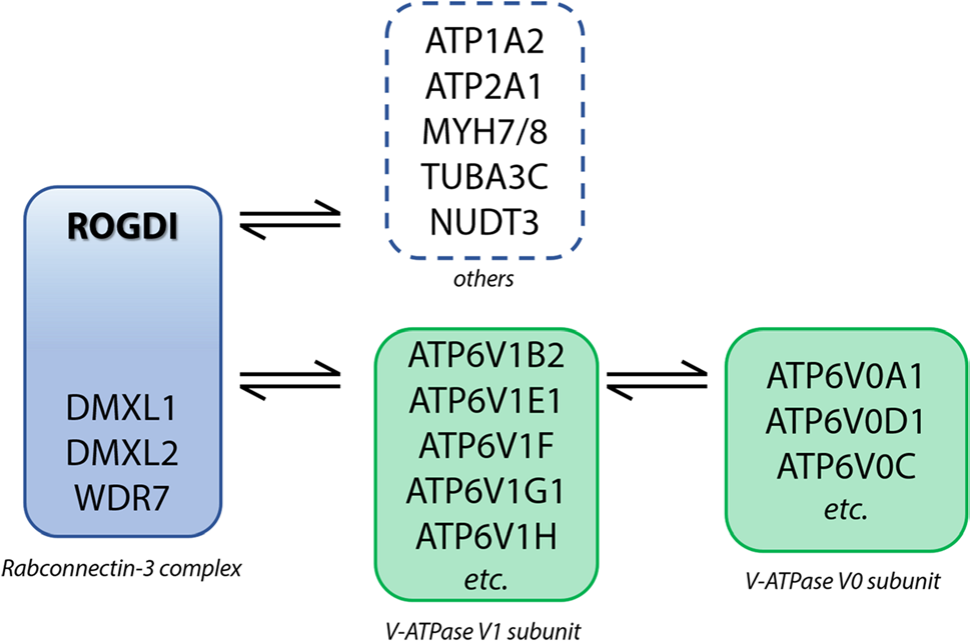
ROGDI protein interaction network. The protein–protein interaction network of ROGDI was assembled based on the results of the OpenCell and BioPlex interactomic projects. ROGDI was found to be part of the Rabconnectin-3 complex, which interacts with the V1 subunit but not with the V0 subunit of the V-ATPase complex. In addition, ROGDI was also found to interact with other partners: ATPase proteins (Atp1a2, Atp2a1), myosin heavy chain (Myh7, Myh8), Tuba3c, and Nudt3.

ROGDI protein interactions (Bioplex and OpenCell interactomes^29,30^) indicated that ROGDI interacts with ATPase proteins (ATP1A2, ATP2A1), myosin heavy chain (MYH7, MYH8), TUBA3C, NUDT3, and 3 proteins of the Rabconnectin-3 complex—WDR7, DMXL1 and DMXL2—(Fig. 6) associated with V-ATPase assembly, which is essential for acidification^26,28^. In humans, it has been reported (BioGRID, https://thebiogrid.org/) that ROGDI interacts with the same proteins described above and subunits of the V1 complex of V-ATPases (ATP6V1G1, ATP6V1E2, ATP6V1H, ATP6V1C1, ATP6V1E1, ATP6V1F and ATP6V1B2). ROGDI also interacts with DISC1 (implicated in schizophrenia and neuronal migration^31^), CEP63 (linked to microcephaly and dyslexia^32,33^), CIT and KIF14 (linked to microcephaly^34^), PLEKHA4 (involved in autism^35^), and KIAA1377 (associated with amyotrophy^36^) (see Supplementary Figure S9).

## Discussion

### The mouse loss of function of *Rogdi* recapitulates major clinical KTS changes

Understanding and finding treatments for extremely rare genetic disorders such as Kohlschütter–Tönz syndrome (of which, to date, only 43 cases have been reported worldwide) requires novel tools and data mining. To date, there is no defined molecular function of the ROGDI protein explaining the unique combination of KTS patient defects. Functions have been hypothesized based on ROGDI presynaptic localization, potentially regulating exocytosis in the developing brain^13^. Crystallographic modeling revealed that ROGDI has α domains adapting leucine ZIPPER-like structures and β domains resembling a claudin-like tight junction protein^3^. Patient mutations are expected to cause premature mRNA degradation by nonsense-mediated decay or dramatically alter protein structure causing a loss of function^6^. Here, we demonstrate that the murine *Rogdi*^*−/−*^ reproduces the KTS phenotype observed in humans.

*Rogdi* mouse mutants are susceptible to developing epilepsy and memory impairment, recapitulating KTS patient clinical findings. The cerebellum plays a leading role in coordination, cognition, and language function^37^. *Rogdi*^*−/−*^ mice display various stage-specific increases in the thickness of the external granular layer (EGL). Early increases in EGL thickness (via augmented EGL proliferation) can produce impaired motor coordination in other models^38^. We observed increases in EGL thickness in both the simplex and paramedian lobules of PN15 *Rogdi*^*−/−*^ mice, whereas no modification was observed in the IGL, suggesting that the migration of granule cells from the external layer to the internal layer was preserved but that postmigration apoptosis was delayed or defective. *Rogdi*^*−/−*^ mice also presented a decreased molecular layer in the simplex lobule, possibly reflecting a growth defect in Purkinje dendritic arborization. In the crus II lobule, the reduction in IGL thickness associated with an increase in EGL thickness indicates a delayed organization characterized by an accumulation of premigratory cells in the EGL and a migration defect to the IGL. The hypertrophy of the molecular layer of the crus II lobule could be explained by postmigratory apoptosis and/or synaptic selection defects. Cerebral CT scans and MRI on selected KTS patients show brain atrophy, including a smaller hippocampus and hypoplasia of the cerebellar vermis^1,6,10,23^. The observed architectural defects could explain the clinical signs of KTS patients. Indeed, simplex and paramedian lobules are involved in sensory-motor functions, while the crus II lobule participates in cognitive functions^39^. Dysregulated growth in both *Rogdi*^*−/−*^ mice and KTS patients suggests a central role in disease etiology. Likewise, *Rogdi*^*−/−*^ mice display decreased hippocampal CA1 stratum oriens thickness, suggesting a growth defect in the basal dendritic arbors of pyramidal cells, potentially impairing memory. RNA-seq supported the dysregulation of genes regulating hippocampal development, synapse organization, and neurotransmitter transport (see Supplementary Table S3).

The mouse model also presents amelogenesis imperfecta with a severe hypomineralization-type enamel phenotype. Mineral content analysis displayed a lack of mineralization in both incisors and molars in the *Rogdi*^*−/−*^ mice with low calcium and phosphate concentrations. Mutants also display higher carbon levels, indicating the presence of organic content^40^. Normally, organic reductions occur between the maturation stage and mineralized enamel. The absence of this reduction in *Rogdi*^*−/−*^ mutants indicates impaired enamel matrix protein degradation and/or removal, which is required during the maturation stage of amelogenesis^41^. Thus, these results confirmed that most mutations affecting *ROGDI* found in individuals with Kohlschütter–Tönz syndrome are loss-of function mutations.

### Alterations in several enamel-regulating genes contribute to the dental phenotype in the ***Rogdi***^*−****/****−*^mutant

During tooth development, enamel may be modified in its width, microstructure, or mineralization degree, causing amelogenesis imperfecta (AI)^42^. To date, variants in over 95 genes are associated with nonsyndromic or syndromic AI^12^. In KTS, enamel is soft, rough, and stained in various shades of brown, presenting AI of the hypomature/hypomineralized type^1,2,6–8^. *Rogdi*^*−/−*^ mouse enamel is chalky, easily chipping and white compared to the normal strong yellow/orange enamel found in wild type mice. Morphologically, both secretory and mature ameloblasts were disorganized, slightly shorter and lacked polarization. *Rogdi*^*−/−*^ defects appear during enamel maturation, showing a lack of mineralization quantified by the reduction of calcium (~ 80%) and phosphorus (~ 50%) in a normally organized nondegraded enamel matrix. Rogdi protein localization in the apical pole of ameloblasts (see Fig. 2) suggests roles in EMP degradation to allow mineralization^41,43^. This severe hypomineralization accompanies defective crystallization. These defects are similar to phenotypes seen in mouse mutants for *Klk4* and *Wdr72*^44–46^.

Whole-transcriptome analysis revealed that the *Rogdi* mutation reduced (in around 50%), transcripts coding for enamel matrix proteins (*Enam, Amelx*, and *Ambn*) and several components of the cell adhesion pathway (see Table 3). *Enam, Amelx*, and *Ambn* are expressed during early stages of amelogenesis^47^ and are essential for controlled enamel crystallite growth and prismatic architecture^43,48,49^. Hu et al.^50^ reported that the thickness of the enamel varies randomly in *Amelx*^+*/−*^ mice.

Other downregulated genes whose mutations produce amelogenesis imperfecta included *Lama3, Lamb3, Lamc2* and *Col17a1* (Table 3). These proteins participate in cell-to-cell or cell-to-extracellular matrix adhesion. Ameloblasts must tightly contact the extracellular matrix^51^. LAMA3, LAMB3 and LAMC2 form the heterotrimeric protein laminin 332 (LM332), located in mature ameloblasts and Tomes’ processes hemidesmosomes, the latter structure mediating cell adhesion^52,53^. COL17A1 is another component of hemidesmosomes and a ligand for LM332, participating in epithelial-mesenchymal interactions required for ameloblast differentiation and adhesion and enamel formation^51^. *Col17−/−* mice, similar to *Rogdi*^*−/−*^ mice, exhibit reduced expression of the enamel proteins Amelx, Ambn, and Enam, further indicating incomplete ameloblast differentiation^54^.

Genes involved in Tomes’ process regulation, vesicle/ion transport, cell adhesion, and pH sensing during enamel maturation are often increased in PN5 *Rogdi* mutant incisors. These include *Amtn, Wdr72, Slc24a4, Cnnm4, Orai1,* and *Gpr68,* which are overexpressed in PN5 incisors. Amelotin (AMTN) may regulate cell–matrix adhesion, promoting enamel crystallization^55^. The enamel of *Amtn*-overexpressing mice has disorganized enamel crystals, which are thin, fragile, and lack Tomes’ processes—a structure marking secretory ameloblasts^56^. WDR72 appears to be required for endocytic vesicle trafficking^44^. Endocytosis is needed to remove degraded enamel protein debris, supporting enamel crystallite growth^51^. Excess WDR72 (found in maturation stage ameloblasts^57,58^) might dysregulate enamel matrix protein processing. Excess WDR72 may contribute to *Rogdi* mutant *Enam, Amelx*, and *Ambn* reductions. The Na^+^/Ca^2+^ + K^+^-Exchanger SLC24A4 is also increased. It has been hypothesized that SLC24A4 is responsible for actively transporting Ca^2+^ ions from ameloblasts into the enamel matrix during maturation^59^. SLC24A4 excess might dysregulate Ca^2+^ levels in *Rogdi* mutant enamel. CNNM4 protein is localized in ameloblasts and mediates transcellular Mg^2+^ transport^60^. *Orai1* forms the pore of the calcium release-activated calcium channel. It has strong expression during the secretory stage, which fades at the end of the maturation stage of amelogenesis^61^. GPR68 is also expressed in ameloblasts through all stages of amelogenesis, with strong expression at the ameloblast apical pole serving as a pH sensor and directing ameloblasts to switch between conformations—ruffle-ended and smooth-ended—at the maturation stage^62^.

While no enormous reductions in gene expression were observed in *Rogdi*^*−/−*^ mutant incisors, cumulative alterations could be synergistic. In humans, *ENAM* variants exhibit dosage-dependent phenotypes^63^. In the case of *AMBN* variants, only biallelic defects will cause amelogenesis imperfecta^64^. Mice lacking a single copy of two enamel-regulating genes, such as *Mmp20/Klk4,* demonstrating smaller reductions in two matrix proteases, can indeed synergize during dental enamel formation^45,49,65^.

Collectively, these results indicate that even though Rogdi is not expressed in the nucleus, the loss of function could be causing expression changes, noting possible indirect effects with Rogdi interacting proteins. Most downregulated genes are expressed during the secretory stage of amelogenesis, controlling ameloblast differentiation and/or adhesion. In contrast, upregulated genes play a more significant role in vesicle/ion transport, cell adhesion, and/or pH sensing during enamel maturation. It is noteworthy that our transcriptomics data suggests that *Rogdi *^*−/−*^ mice may also display hypoplastic enamel; this could be further investigated^66^.

### *Rogdi* mutants can be used to understand many KTS-associated features

According to our data, the *Rogdi* mutant mouse model presents a susceptibility to epilepsy. Behavioral test results indeed suggest that *Rogdi*^*−/−*^ hyperactivity and memory defects reflected intellectual impairments found in KTS^1,2,6^. Some *Rogdi*^*−/−*^ female mice also displayed decreased muscle strength. These data correlate with KTS patient phenotypes. The *Rogdi* mutant mouse exhibits severe enamel defects in both incisor and molar teeth. *Rogdi* mutants form less mineralized enamel, producing an “amelogenesis imperfecta”-like phenotype, perfectly mimicking severe KTS patient defects.

In addition to the typical KTS features, *Rogdi* mutants also present problems associated with the digestive system, which is potentially linked to pH regulation. ROGDI is expressed in the digestive organs^67^. Investigating whether digestive disorders are present is indeed relevant for overall patient quality of life. This model could help to decipher new symptoms and improve patient treatment.

Concerning the antiepileptic treatments used in patients, described that therapy with perampanel gives good results^9^. Perampanel (PER) is a first-in-class orally active, selective, noncompetitive alpha-amino-3-hydroxy-5-methyl-4-isoxazolepropionic acid (AMPA) receptor agonist^68^. AMPA receptors are hypothesized to be involved in the synchronization of excitatory glutamatergic transmission, which leads to the production of seizures. Perampanel had a small but discernible impact on NMDA receptors when tested in vitro. PER was found to be more effective at preventing seizures in mouse models of tonic–clonic generalized seizures and absence seizures than carbamazepine and sodium valproate^69^. Other treatments have been used in KTS patients without much success^2,10,70^. As *Rogdi*^*−/−*^ has epilepsy susceptibility, it could serve as a model to test new antiepileptic drugs for KTS patients and their mechanism of action.

### ROGDI bridges with the V-ATPase pathway, a link between enamel formation and other functions

Loss of *ROGDI* might produce enamel and brain defects by perturbing the vacuolar type (V)-ATPase-dependent lysosomal acidification pathway^71^. V-ATPases (H^+^-ATPases) are multisubunit, ATP-dependent proton pumps made up of an integral membrane V0 subcomplex that creates the transmembrane proton pore and a peripheral V1 subcomplex that contains the sites of ATP hydrolysis. They control pH homeostasis, participate in vesicle transport and membrane fusion, and play crucial roles in synaptic transmission^26^. Defects in V-ATPase action impair the ATP-driven proton pump, disrupting lysosomal acidification. This contributes to lysosomal storage disorders, a family of disorders marked by severe neurodegeneration, epilepsy, alterations found in Alzheimer’s disease and Parkinson’s disease^72^, and to other neurodevelopmental/neurodegenerative diseases^73^. V-ATPase function is indispensable for viable adults^74^. V-ATPase function is essentially required to maintain lysosomal pH gradients, a process also required in tooth formation. Missense mutation of the V-ATPase component *ATP6V1A* produces enamel dysplasia in patients, along with epilepsy and brain atrophy^75^. V-ATPase a3 subunit disruption in mice can cause hypoplastic/hypomineralized enamel due to disruptions in the secretory stage of amelogenesis^76^.

We hypothesized that the unique combination of neurological and enamel phenotypes characterizing KTS patients could result from defective V-ATPase-driven acidification. The V1 subcomplex can be released from the V0 subcomplex in response to signals such as glucose deprivation, inhibiting ATPase activity and proton transport. The disassembly of V-ATPase is a mechanism that regulates ATPase activity. Assembly of V1 with V0 (required for ATP-driven proton transport) requires assistance of the yeast RAVE (Regulator of H^+^-ATPase of Vacuolar and Endosomal membranes) or its functional equivalent in higher eukaryotes—the Rabconnectin-3 complexes^28^. The aldolase enzyme also participates in V-ATPase assembly. Aldolase mutant cells have a phenotype comparable to the Rav1—a RAVE component-mutant. It has been reported that the RAVE complex and aldolase may work together to control V-ATPase assembly^77^.

The crystal structure of one of the RAVE complex components, Rav2, is strikingly similar in its domains to the human ROGDI protein^28^. The interactome of human ROGDI was explored in OpenCell^30^. Several endogenously tagged proteins from the V1 subunit of the V-ATPase complex were able to identify ROGDI as an interaction partner. It was also found that ROGDI shares an interactomic community with DMXL1, DMXL2, and WDR7, components of the V-ATPase chaperone Rabconnectin-3 complex. Despite their high interactomic similarity, no direct interactions could be detected between them, possibly because all these proteins displayed very low cellular abundance, making their endogenous complexes challenging to capture. However, by overexpressing DMXL1 or WDR7, it was possible to detect their interaction with ROGDI. A similar conclusion was observed in the BioPlex interactome project (Supplementary Figure S9A), where overexpressed ROGDI was found to interact with other components of the Rabconnectin-3 complex (DMXL1, DMXL2, and WDR7), among others^78^. In addition, all Rabconnectin-3 complex components (Rogdi, Dmxl1, Dmxl2 and Wdr7) were identified as interaction partners of the V-ATPase B1 subunit based on immunoprecipitation in mouse kidney lysates^26^.

Our results solidify the ROGDI-V-ATPase connection. *Rogdi*^*−/−*^ mutants exhibit enamel acidification impairment (see Fig. 5D), likely accounting for morphological deterioration in enamel structure. RNA-seq data from *Rogdi* mutant incisors shows increased *Slc9a3r2*, *Atp6v0c* and *Wdr72* (a WDR7 homologue) levels -known to interact with the acidifying V-ATPase complex and with DMXL1 and DMXL2—proteins interacting with Rabconnectin-3 complex (also required for acidification). These changes likely affect pH regulation, disrupting acidification^57^. Given the similarity to WDR7 and links to pH control, it is important to investigate the possibility that WDR72 may participate in Rabconnectin-3 complexes, at least in some specific locations such as teeth. *Rogdi*^*−/−*^ mutants can indeed be used to explore whether a reduction in lysosomal acidification in brain sites such as the hippocampus explains defects in learning in *Rogdi*^*−/−*^ mutants in future studies. Along with lysosomal defects, reduced glutamate uptake due to pH imbalance can also lead to neurodegenerative diseases^79^. Indeed, V-ATPase pumps regulate pH in the hippocampus and have been implicated in Alzheimer’s and Parkinson’s disease^80^. Here, V-ATPase and *DMXL2* reductions can also produce epilepsy^27,81,82^. V-ATPases have a role in the filling and exocytosis of glutamate, helping in the acidification of the vesicle and facilitating membrane binding of the vesicle followed by glutamate exocytosis by detachment of the V1 and V1C1 domains from the V0 domain^79^.

In the stomach, V-ATPase may serve as an additional pathway for acid secretion or as an internal proton-buffering mechanism. It has been reported that mice lacking functional V-ATPase (ATP6v0a3) in the stomach have an elevated gastric pH^83,84^, which may also help to explain the stomach phenotype found in *Rogdi*^*−/−*^ mutants, where pH also appears to be higher in *Rogdi*^*−/−*^ compared to WT (Fig. 3C).

The acidic pH on the surface of maturing enamel is probably due to the crystallization of hydroxyapatite (HA), since the precipitation of HA produces a considerable number of protons^85^.Although it is unlikely that ameloblasts pump protons into extracellular matrix using V-ATPases, V-ATPase significantly increases during the maturation stage at the apical membrane of ameloblasts compared to the secretory stage^86^.

Enamel grows in a pH-sensitive protein matrix that is related to enamel protein self-assembly and enamel crystal development^87^. During modulation, the matrix is broken down, and fragments are endocytosed by maturation ameloblasts and/or papillary cells^41^.We show an absence of acidification in mutant incisors (Fig. 5D), suggesting that the absent pH reductions in *Rogdi* mutants blocked the ameloblast maturation stage by hindering the stepwise removal of EMPs by endocytosis such as amelogenins from the crystal surface of enamel^41,88^. Defects in ameloblast organelle acidification that limit the degradation of enamel proteins can indeed cause amelogenesis imperfecta^41,89^. When pH modulation is delayed or disrupted, enamel mineralization is known to be reduced^90,91^. This defect in acidification is observed in *Rogdi*^*−/−*^ mutants (Fig. 5D).

V-ATPase alterations can also aggravate or produce cancer, neurodegenerative diseases, and diabetes and disrupt energy and nutrient-sensing functions within cells^92^. People with KTS present epilepsy, psychomotor regression, intellectual disability (ID), nephrocalcinosis, and enamel defects^9^. Distal renal tubular acidosis is also linked to mutations in V-ATPase subunit isoforms and could lead to nephrocalcinosis^28,93^. Neurons similarly use energy from proton-pumping V-ATPases to load neurotransmitters into synaptic vesicles. Synaptic signal propagation requires neurotransmitter release^94^; hence, V-ATPase or other acidification disruptions could produce a range of ID, epilepsy, or hyperactivity symptoms^73^. Considering ROGDI as a regulator of V-ATPase assembly, it could be interesting to study treatments targeting the reassembly of the proton pump to help in its function.

The *Rogdi* mutant will be a useful new model for analyzing treatment outcomes, according to all the findings provided here. Hence, these findings point to new perspectives on the function of ROGDI in cell biology and pathophysiology.

## Material and methods

### Animals

The *Rogdi* cKO mutant mouse line was established at the Mouse Clinical Institute (Institut Clinique de la Souris, MCI/ICS)—PHENOMIN (http://www.phenomin.fr) in the Genetic Engineering and Model Validation Department.

The targeting vector was constructed as follows. A 2.8 kb fragment encompassing exons 6 (ENSMUSE00000128063) to 11 (ENSMUSE00000427909) comprising the whole interLoxP genomic sequence was amplified by PCR (from BAC RP24-424L20 genomic DNA) and subcloned in an MCI proprietary vector. This ICS vector includes a neomycin resistance cassette surrounded by FRT sites, as well as 2 loxP sites. Two PCR fragments corresponding to the 5’ and 3’ homology arms (1.8 and 1.7 kb, respectively) were subsequently cloned into the plasmid obtained in the first cloning step to obtain the final targeting construct. The linearized construct was electroporated into C57BL/6N mouse embryonic stem cells (ESCs; S3 in house developed line). After G418 selection, targeted clones were identified by PCR using external primers and further confirmed by Southern blotting with a Neo probe (5’ and 3’ digests) as well as a 3’ external probe. Four positive ES clones were injected into BALB/cN blastocysts. The resulting male chimeras were bred with Flp deleter females that show maternal contribution^95^. Germline transmission of the cKO allele with direct excision of the flipped selection cassette was obtained. The resulting line was mated with ROSA26-deleter CRE mice to delete exon 6 to exon 11. Offspring mice were backcrossed on a C57BL/6N background.

*Rogdi*^*−/−*^ mice were genotyped with oligonucleotides and PCR amplification as described in Supplementary Figure S1. The mice were fed ad libitum with humidified pellets placed at the bottom of the cage. *The Rogdi* gene targeting strategy, as well as the characterization of mice with a disrupted *Rogdi* gene, are illustrated in Fig. 3 and Supplementary Figure S1.

### In situ hybridization

A full-length *Rogdi* probe (Dharmacon) was used to generate an antisense probe. In situ hybridization (ISH) was performed using digoxigenin-labeled RNA probes on 10 μm frozen sections. The samples were fixed in 4% paraformaldehyde for 10 min at 4 °C, rinsed with PBS, dehydrated in graded ethanol (70%, 95%, 100%, and 95%) for 5 min each, and dried for 1 h. The probe was diluted to 1 µg/ml in prewarmed hybridization buffer (65 °C) and denatured for 10 min at 70 °C. The slides with the probe and coverslip were placed in a humidified chamber at 65 °C overnight. 5 × SSC prewarmed (70 °C) was used to allow coverslips to detach. The samples were washed for 1 h at 70 °C in 0.2 × SSC and transferred to 0.2 × SSC room temperature (RT) for 15 min. Washing for 2 × 30 min at RT with slow agitation in 1 × maleic acid buffer Tween (MABT) was followed by blocking in MABT with 2% blocking reagent and 20% heat-inactivated normal goat serum (NGS) for 1 h at RT, and a 1/2000 anti-digoxigenin antibody in blocking solution was added for 2 h at RT. Washing 5 × 20 min at RT in MABT 1x, rinsing 2 × 10 min in alkaline phosphatase buffer (NTMT) 1 × was performed. Hybridized probes were visualized with NTMT, BCIP and NBT in PBT.

### Immunohistochemistry

Embryos (E12.5 to E18.5) and postnatal day (PN) 1, 3, 5, 7 and 14 heads were freshly fixed in 4% paraformaldehyde for 24 h, demineralized with 10% EDTA (postnatal stages), cryoprotected with 20% sucrose, and embedded in Shandon Cryomatrix Frozen Embedding Medium (Thermo Scientific™). Frozen sagittal Sects. (10 μm) were cut using a Leica CM3050 S cryostat and placed on Superfrost Plus™ slides for immunohistochemistry. Antigen retrieval was performed according to the manufacturer’s antibody protocol. The samples were blocked with 5% normal donkey serum (NDS) or normal goat serum (NGS) in 0,05% TBS Tween 20 (TBSTw). Sections were incubated with anti-Rogdi (Proteintech^®^ 17047-1-AP) in blocking solution (1:100). After washing with TBSTw, samples were incubated with secondary Ab labeled with fluorophore in TBS (1:500) and DAPI (5 mg/ml) to a final dilution of 1:5000–10,000. Sections were mounted with FluoroMount-G Mounting medium (FP-483331, Interchim). Images were acquired with an upright motorized microscope (Leica DM 4000 B) equipped with a Photometrics CoolSNAP HQ2 camera and analyzed in Leica Application Suite X software.

### Data analysis of single-cell sequencing of the central nervous system

Single-cell transcriptomics of twenty-four cell populations in five regions of the central nervous system produced by^16^ were analyzed for *Rogdi* expression. Reads were grouped into low (50 RPKM to 500 RPKM), moderate (500 RPMK to 2000 RPKM), or high expression (> 2000 RPKM).

### Data analysis of single-cell sequencing of the incisor epithelium

*Rogdi* expression in incisor epithelium was analyzed in https://kleintools.hms.harvard.edu/tools/springViewer_1_6_dev.html?datasets/Sharir_et_al_2019/control_epithelial. This tool identifies distinct cell types by single-cell transcriptomic analysis and delineates their spatial organization in the incisor epithelium. SPRING representation of the incisor epithelium dataset described in^17^ presents 15 spectral clusters, grouped into 3 main classes: cycling cells, pre-ameloblasts and ameloblasts, and non-ameloblast epithelial cells.

### Behavioral tests

WT (n = 16 [first cohort = 6 males and 3 females/second cohort = 3 males and 4 females]) and *Rogdi*^*−/−*^ (n = 14 [first cohort = 3 males and 3 females/second cohort = 3 males and 5 females]) mice at 10 weeks of age at the beginning of the study were tested. Different tests to check activity, memory and locomotor activity were performed (circadian activity, elevated plus maze, novel object recognition, gross neurological examination, grip, rotarod). Data from 2 independent batches and from both sexes were pooled when no batch effect or sex effect was detected.

### Gross neurological examination (SHIRPA)

General health and basic sensory motor functions were evaluated using a modified SHIRPA protocol. This analysis provided an overview of physical appearance, body weight, neurological reflexes, and sensory-motor abilities. Data were analyzed using unpaired Student’s t-test.

### Circadian activity

Spontaneous locomotor activity and rearing activity were measured using individual cages (20 × 10 × 8 cm) equipped with infrared captors (Imetronic, Pessac, France). Mice were tested for 32 h to measure habituation to the apparatus as well as nocturnal and diurnal activities. The results were expressed per 1 h period and/or as a total of the different activities. Statistical analyses were performed using two-way ANOVA, Sidak’s multiple comparisons and unpaired Student’s t-test.

### Novel object recognition

Mice were evaluated in a circular closed arena (30 cm diameter and 50 cm height basin). An EthoVision XT video tracking system (Noldus, Wageningen, Netherlands) was used to record locomotor activity. The arena was homogeneously illuminated at 20 Lux. Animals spent 10 min habituating to the arena. Each mouse was placed in the periphery of the arena and given full reign to freely explore the apparatus. The distance travelled was recorded over the test session. The following day, mice were tested for object recognition in the same arena. They underwent a 10-min acquisition trial during which they were positioned in the arena in the presence of two test objects (A and A'). The time the animal spent exploring the samples (sniffing) was manually recorded. One hour later, a 10-min retention trial was conducted. The animal was placed in the arena with one of the samples A and an additional object B, and the times tA and tB it took to investigate each item were noted. A recognition index (RI) was determined as (tB / (tA + tB)) × 100. Animals were isolated during the retention interval. Data were analyzed using unpaired Student’s t-test for retention of object exploration and retention index. The Mann–Whitney test was used for the distance travelled during habituation, acquisition and retention sessions and for acquisition object exploration.

### Grip test

This test measures the maximal muscle strength using an isometric dynamometer connected to a grid. Once the animal is holding the grid with its four paws, it is slowly moved backwards until it releases it. Mice were given three testing trials separated by approximately 10 s intervals. The strength developed by the animal was measured in grams and adjusted to the body weight of the animal. Data were analyzed using unpaired Student’s t-test.

### Rotarod test

This test evaluates an animal's ability to stay balanced on a rotating rod (Bioseb, Chaville, France). Mice were given three testing trials during which the rotation speed accelerated from 4 to 40 rotations per minute in 5 min. Trials were separated by 15 min intervals. Endurance and motor coordination performance were measured using the average latency. Data were analyzed using unpaired Student’s t-test.

### Elevated plus maze

The apparatus used is automated and made of PVC (Imetronic, Pessac, France). It consists of two open arms (30 X 5 cm) opposite one to the other and crossed by two enclosed arms (30 × 5 × 15 cm). The apparatus is equipped with infrared captors allowing the detection of the mouse in the enclosed arms and different areas of the open arms. Mice were tested for 5 min, during which the number of entries into and time spent in the open arms were measured and used as an index of anxiety. Closed arm entries and total arm entries were used as measures of general motor activity. Data were analyzed using unpaired Student’s t-test.

### Muscle analysis

The tibialis anterior, gastrocnemius and quadriceps muscles were dissected from 8-week-old WT (n = 3) and *Rogdi*^*−/−*^ (n = 3) mice and used for light microscopy and scanning electron microscopy (SEM). Muscle weight was measured and normalized to body weight. For light microscopy, 10 µm cross-sections of snap-frozen muscle were stained with hematoxylin and eosin and Gomori trichrome. Stained sections were digitalized with a NanoZoomer 2.0-HT (Hamamatsu Photonics). Histological analysis was performed using NDP.view2 software. For SEM, ultrathin serial sections were picked up on grids and contrasted with uranyl acetate and lead citrate. The grids were examined for morphology analysis with a Morgagni 268D electron microscope (FEI Electron Optics, Eindhoven, the Netherlands) equipped with a Mega View III camera (Soft Imaging System). Muscle fiber size analysis was performed in the tibialis anterior, including an average of 1986 (range 1380–3499) muscle fibers per individual. Data from 6 mice with a total of 11,914 fibers were acquired and analyzed. Fiji and Cellpose 2.0 software were employed for the study. The fibers’ smallest diameter i.e. The minimum diameter of Feret (MinFeret) was determined (fibers located at the periphery of the sections not fully captured in the photographs were excluded from the analysis). Normally distributed continuous data for muscle ratio/body weight and small fiber percentages were summarized as the means ± standard deviation. Prism 9 (GraphPad Software, San Diego, CA) was used for data analysis using unpaired Student’s t-test and Welch’s correction when unequal standard deviations were found.

### Pentylenetetrazol induced seizure (PTZ)

WT (n = 8) and *Rogdi*^*−/−*^ (n = 7) males, aged between 10 ± 1 weeks, were used to test epilepsy susceptibility. Pentylenetetrazol is a noncompetitive GABA-A receptor antagonist that induces seizures and is used to determine the seizure threshold. The animal was intraperitoneally injected with 40 mg/kg pentylenetetrazol and placed in a translucent cage. The different stages of the seizure that the animal exhibited were observed during the 20 min after injection.

### Cerebellum and hippocampal formation analysis

Postnatal day (PN) 15 brains (n = 6 for *Rogdi*^*−/−*^ and n = 6 for WT) were rapidly removed after decapitation and snap-frozen in cooled (−35 °C to − 45 °C) 2-methylbutane. Brain samples were stored at − 80 °C. Frozen coronal sections (20 µm) were fixed with 4% formaldehyde solution for 24 h and stained with 0.5% cresyl violet (Sigma-Aldrich, France) solution following the protocol proposed by Bolam (1992)^96^. Slides were mounted with mounting medium (Eukitt^®^). Cerebellum and hippocampus images were digitalized with NanoZoomer 2.0-HT (Hamamatsu Photonics). Structures of interest were identified using the Franklin and Paxinos mouse brain stereotaxic atlas (third edition, Elsevier 2007)^97^. The thicknesses of the internal and external granular layers (IGL and EGL, respectively) and the molecular layer (Mol) were measured using NDP.view2 (Hamamatsu) software. Planimetry of the cerebellar cortex on the sections was verified by observing that Purkinje cells formed a continuous line parallel to the lobule fissure^98,99^. In a central segment of each lobule, six measurements per section from two sections per mouse were performed. The Purkinje cell layer made up of perikaryal was included in the molecular layer. All selected lobules were measured posterior to the bregma point, at a level of 6.12 mm for simplex and vermis IV-V, at a level of 6.64 to 6.84 mm for paramedian and at a level of 6.36 to 6.72 for crus I and crus II. The CA1 and dentate gyrus granular and molecular layers of the hippocampal formation were measured in a similar manner at 1.7 to 1.82 mm posterior to the bregma point. Statistical analysis was performed using Student’s t-test.

### Blood analysis

Blood was collected at the temporal vein on unfasted conscious WT (n = 9, 6 males and 3 females) and *Rogdi*^−/−^ (n = 5, 3 males and 2 females) mice at the age of 14 weeks. Blood chemistry was performed on an OLYMPUS AU-480 automated laboratory workstation (Beckmann Coulter, US) with kits and controls supplied by Beckmann Coulter. Internal quality control materials (Olympus) were analyzed daily to monitor precision throughout the experiment. Blood chemistry parameters were measured on the plasma samples: calcium, phosphorus, and alkaline phosphatase (ALP). A complete blood cell count was performed on total blood using the Veterinary hematology analyzer Element HT5 (Scil Animal Care, France). Statistical analysis was performed using Student’s t-test.

### Microtomography (µ‐CT) imaging

The heads of 20 8-week-old adult (10 females and 10 males) *Rogdi‐/‐* mutant and wild-type (WT) mice were analyzed. All samples were scanned using the Quantum FX micro-CT imaging system (Caliper Life Sciences, Hopkinton, MA, USA), which operates at 80 kV and 160 μA, with high resolution (pixel size of 10–80 μm), to evaluate the morphology and density of the skull and tooth. A 3-D model was created using the software Slicer 4.10.2. Dento-cranio-facial bone anatomy was sorted into coronal, transverse, and sagittal planes at the levels of the skull, mandible, and teeth. The images were analyzed using FIJI (ImageJ2), winEDMA, MorphoJ 1.07.a and Stratovan Checkpoint (Stratovan Corporation. Version 2018.08.07). Density was measured using a minimum of 3400 Hounsfield units (HnsfU), a relative quantitative measurement of radiodensity.

### Scanning electron microscopy (SEM)

Mandibles of 8-week-old control and *Rogdi*^*−/−*^ mice were dissected and stored in 70% ethanol at 4 °C. Samples were embedded in Epon 812 (Euromedex, Souffelweyeresheim, France) and then sectioned along the sagittal plane using a diamond saw mounted on a microtome (Walter EBNER, Le Locle, Switzerland). The sample surface was polished with 1200, 2400, and 4000 SiC abrasive papers under continuous water irrigation^100^. Then, the polished sections were etched using 20% citric acid for 2 min, rinsed for 10 s with distilled water, and dehydrated in a graded series of ethanol solutions. Mounting was performed on aluminum SEM stubs and sputter-coated with a gold–palladium alloy (20/80 weight %) employing a Hummer JR sputtering device (Technics, CA, USA). The morphological and chemical characteristics were determined using a Quanta 250 FEG scanning electron microscope (FEI Com-190 pany, Eindhoven, The Netherlands) at 7.5 kV acceleration voltage of electrons. EDX analysis was performed with a working distance of 10 mm and an acquisition period of 30 s^101^. Measurements were performed on mineralized enamel of the cervical part near the alveolar bone, and for the maturation stage at 2 mm from the cervical loop. The weight percentages of chemical elements on the surfaces of the different dental tissues were attained.

### Methyl red staining

Twelve-week-old hemimandibles from *Rogdi*^*−/−*^ and WT mice were dissected. Bone and enamel organs covering the lower incisors were removed, and cellular debris was cleaned from the enamel surfaces. Fresh filtered methyl red solution (Methyl red-C.I. 13020-50 mg, 0.1 M sodium hydroxide 1.86 mL, 95% ethanol 50 mL, distilled H_2_O q.s. 100 mL) was prepared before dissection. Incisors were dipped into the pH indicator methyl red for at least 10 min. Incisors were placed on filter paper, and pictures were taken immediately after incubation using a Leica M80 stereo microscope equipped with a Motic Moticam 580 camera.

### RNA sequencing

Total RNA was extracted from lower incisors of 6 males per genotype, control and *Rogdi*^*−/−*^ at PN5, and final analyses were performed with 4 control and 5 *Rogdi*^*−/−*^ samples according to the principal component analysis. Both lower incisors were carefully dissected and processed for RNA extraction using an RNeasy Plus Micro Kit (Qiagen). Gene expression quantification was performed from uniquely aligned reads using htseq-count^102^ version 0.6.1p1, with annotations from Ensembl version 93 genome browser and “union” mode explained in http://htseq.readthedocs.io/en/master/. Only nonambiguously assigned reads were retained for further analyses. Comparisons of interest (WT vs*. Rogdi*^*−/−*^) were performed using the test for differential expression proposed by Love et al.^103^ and implemented in the Bioconductor package DESeq2 version 1.16.1. Genes with high Cook’s distance were filtered out. Independent filtering based on the mean of normalized counts was performed to filter out those genes that had no or little chance of showing significant evidence of differential expression (without looking at their statistic). *p*-Values were adjusted using the Benjamini and Hochberg^104^ method for multiple testing. Significant genes were selected using an adjusted *p*-value < 0.05 and an absolute value of log2 fold-change > 0.3. Data were analyzed using Cytoscape 3.9.1^105^ and DAVID^106^.

### Quantitative real-time PCR (RT-qPCR)

First-strand cDNA synthesis from WT and *Rogdi*^*−/−*^ male lower incisor mRNA was performed with SuperScript^®^ IV Reverse Transcriptase (Invitrogen). LightCycler^®^ 480 SYBR Green I Master (Roche Life Science) incorporation into amplified PCR products was detected using a RealPlex 2 qPCR Real Time PCR ThermoCycler. Primer sequences (listed in Supplementary Table S4) were designed using the Primer3web program. Expression was normalized to glyceraldehyde-3-phosphate dehydrogenase (*Gapdh*) levels. Six mice of each genotype were used to check *Rogdi* exon expression. Tests were performed in triplicate to confirm variations. Statistical analysis was performed by Student's t test.

### Rogdi protein interactions network

Three databases were consulted to look for Rogdi protein interactions:

BioPlex Interactome is a catalog of human protein–protein interactions that BioPlex v3 contains interactomes of 10,128 human proteins from 293 T cells and the interactomes of 5522 proteins from HCT116 cells^29,78^.

OpenCell is a proteome-scale collection of protein localization and interaction measurements in human cells. It contains the interactomes of 1310 endogenous proteins^30^.

A repository for biomedical interactions known as BioGRID (version 4.4.218)^107^ contains data that have been gathered through intensive curation efforts. From 81,725 publications from important model organism species, the data set contains interactions, chemical associations, and post-translational modifications (PTMs). All information was freely accessible for download.

All information was freely accessible for download.

### Ethics statement

All animals were maintained and manipulated under animal protocols in accordance with the French Ministry of Agriculture guidelines for the use of laboratory animals (C67-218-37-IGBMC « Mécanismes responsables de malformations osseuses et bucco dentaires: analyse de modèles murins » n° APAFIS#3957-2016020516359388v1) and with NIH guidelines provided in the Guide for the Care and Use of Laboratory Animals. All methods and experimental procedures were reviewed and approved by the IGBMC institutional safety committee. This study is reported in accordance with ARRIVE guidelines.

### Supplementary Information

Supplementary Information.

## Data Availability

The analyzed RNA sequencing data that support the findings of this study is available in Gene Expression Omnibus with the accession number GSE239863 https://www.ncbi.nlm.nih.gov/geo/query/acc.cgi?acc=GSE239863.
