# Supplementary material for: The Rogdi knockout mouse is a model for Kohlschütter–Tönz syndrome
Source: Sci Rep. 2024 Jan 3;14:445. doi: 10.1038/s41598-023-50870-2 (PMC10764811; doi:10.1038/s41598-023-50870-2)
Supplement: Supplementary file 1 — Supplementary Information. [file 41598_2023_50870_MOESM1_ESM.docx]

**Supplementary material**

The *Rogdi* Knockout Mouse is a Model for Kohlschütter–Tönz Syndrome

**Alexandra Jimenez-Armijo^1^, Supawich Morkmued^2^, José Tomás Ahumada^1^, Naji Kharouf^3^, Yvan de Feraudy^1,4^, Gergo Gogl^1^, Fabrice Riet^5^, Karen Niederreither^1^, Jocelyn Laporte^1^, Marie Christine Birling^5^, Mohammed Selloum^5^, Yann Herault^1,5^, Magali Hernandez^6^, Agnès Bloch-Zupan*^1,7,8,9,10^**

Affiliations

1. Université de Strasbourg, Institut de Génétique et de Biologie Moléculaire et Cellulaire (IGBMC), INSERM U1258, CNRS- UMR7104, Illkirch, France.
2. Faculty of Dentistry, Pediatrics Division, Department of Preventive Dentistry, Khon Kaen University, Khon Kaen, Thailand
3. Université de Strasbourg, Laboratoire de Biomatériaux et Bioingénierie, Inserm UMR_S 1121, Strasbourg, France.
4. Department of Neuropediatrics, Strasbourg University Hospital, Strasbourg, France.
5. Université de Strasbourg, CNRS, INSERM, CELPHEDIA, PHENOMIN, Institut Clinique de la Souris (ICS), Illkirch, France.
6. Centre Hospitalier Régional Universitaire de Nancy, Université de Lorraine, Competence Center for Rare Oral and Dental Diseases, Nancy, France.
7. Université de Strasbourg, Faculté de Chirurgie Dentaire, Strasbourg, France.
8. Université de Strasbourg, Institut d'études avancées (USIAS), Strasbourg, France.
9. Hôpitaux Universitaires de Strasbourg (HUS), Pôle de Médecine et Chirurgie Bucco-dentaires, Hôpital Civil, Centre de référence des maladies rares orales et dentaires, O-Rares, Filière Santé Maladies rares TETE COU, European Reference Network ERN CRANIO, Strasbourg, France.
10. Eastman Dental Institute, University College London, London, United Kingdom.

***Corresponding author**

Agnès Bloch-Zupan

agnes.bloch-zupan@unistra.fr


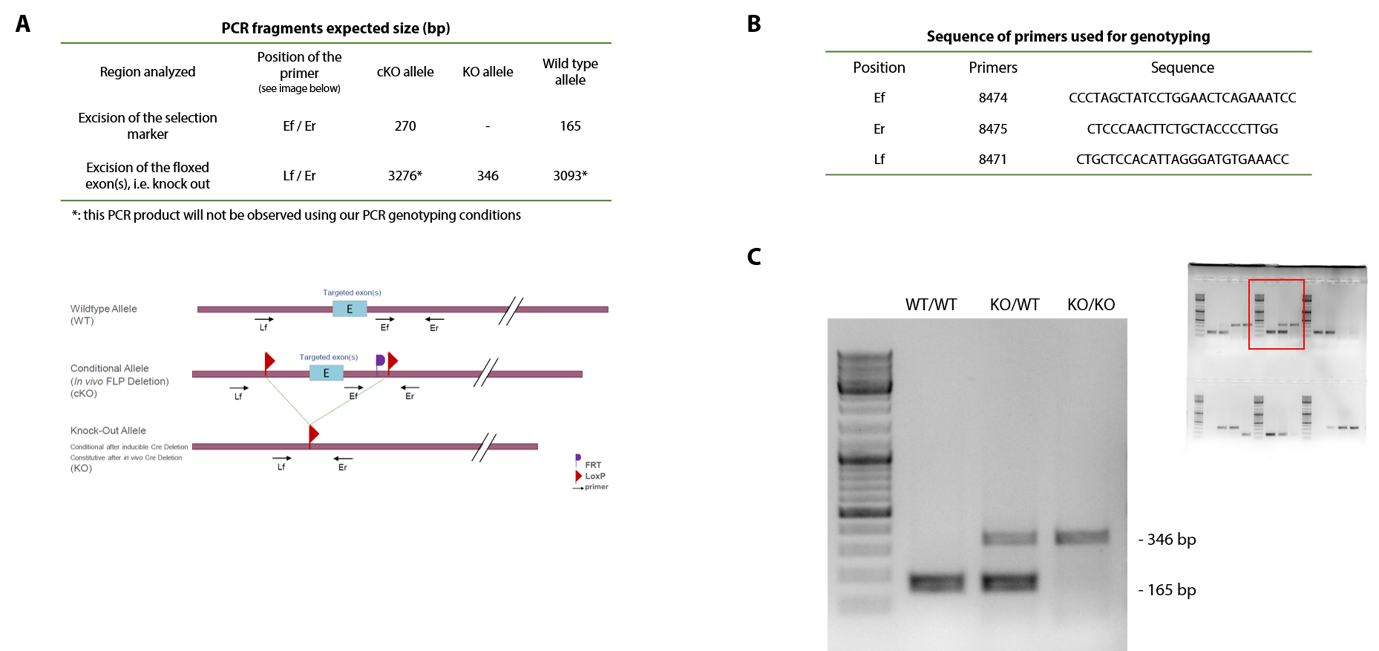


**Supplementary Figure S1. Rogdi knockout genotyping strategy.** (A) Table shows PCR fragments expected size of the different regions analyzed. Knock out strategy for deletion of exons 6 to 11 prior to and after homologous recombination is illustrated. Arrows identify primer annealing sites for RT‐PCR. (B) Primers sequence used for genotyping. (C) Shows an example of PCR genotyping of littermates from heterozygous breeding. The lower bands (165 bp) are the wild‐type amplification products; the higher bands (346 bp) are the same products lacking exon 6 to 11. At right, the original gel image, indicating with a red box the example.

**
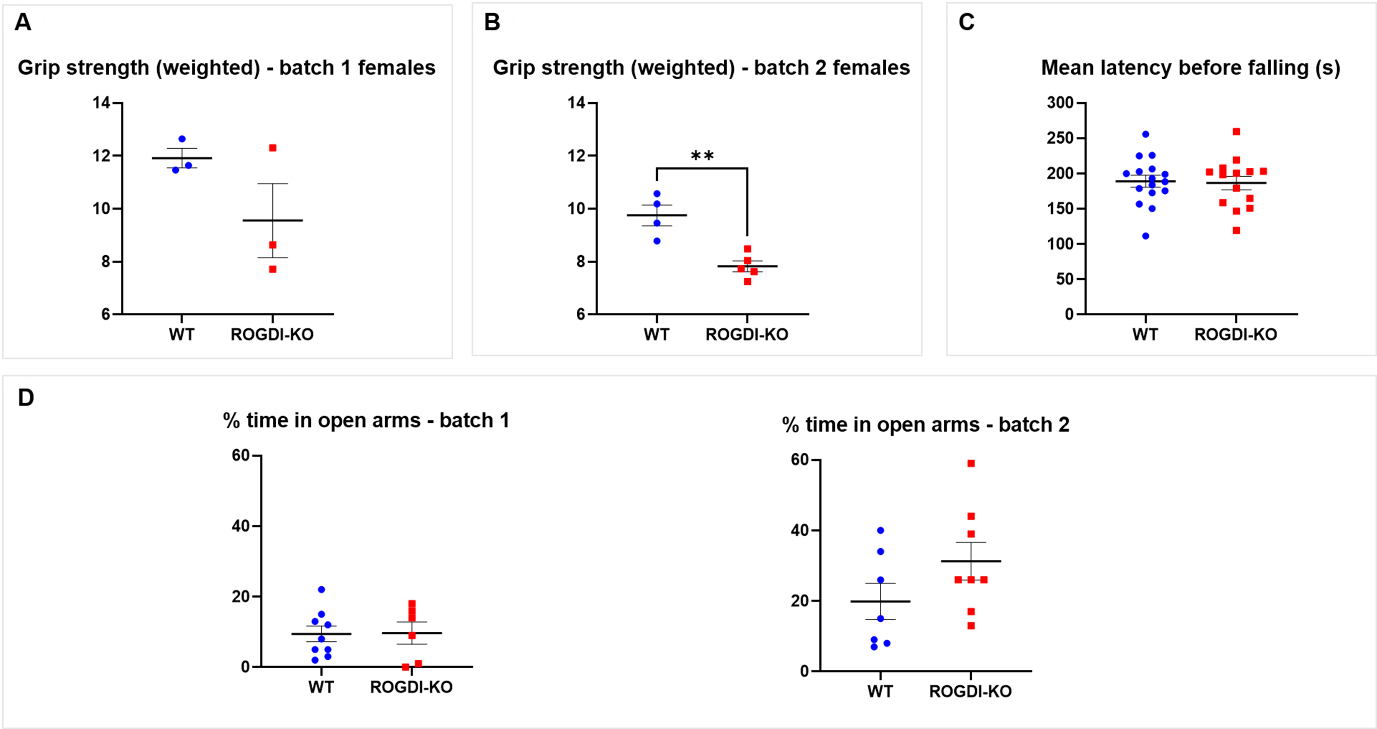
**

**Supplementary Figure S2. Skeletal muscle function, motor coordination and balance, and anxiety-related behavior in *Rogdi^-/-^* mice.** (A, B) Raw grip strength (4 paws) and grip strength adjusted to body weight (mean of 3 trials), second batch (B) of females show a significant decrease in muscle strength compared to WT. (C) Latency before falling from the rotarod accelerating from 4 to 40 rpm in 5 min (mean of the 3 trials) shows no difference between genotypes. (D) Percentage of time spent in the open arms for batch 1 and batch 2 show no difference between groups. Data are expressed as mean ± SEM. ** p < 0.01.

**
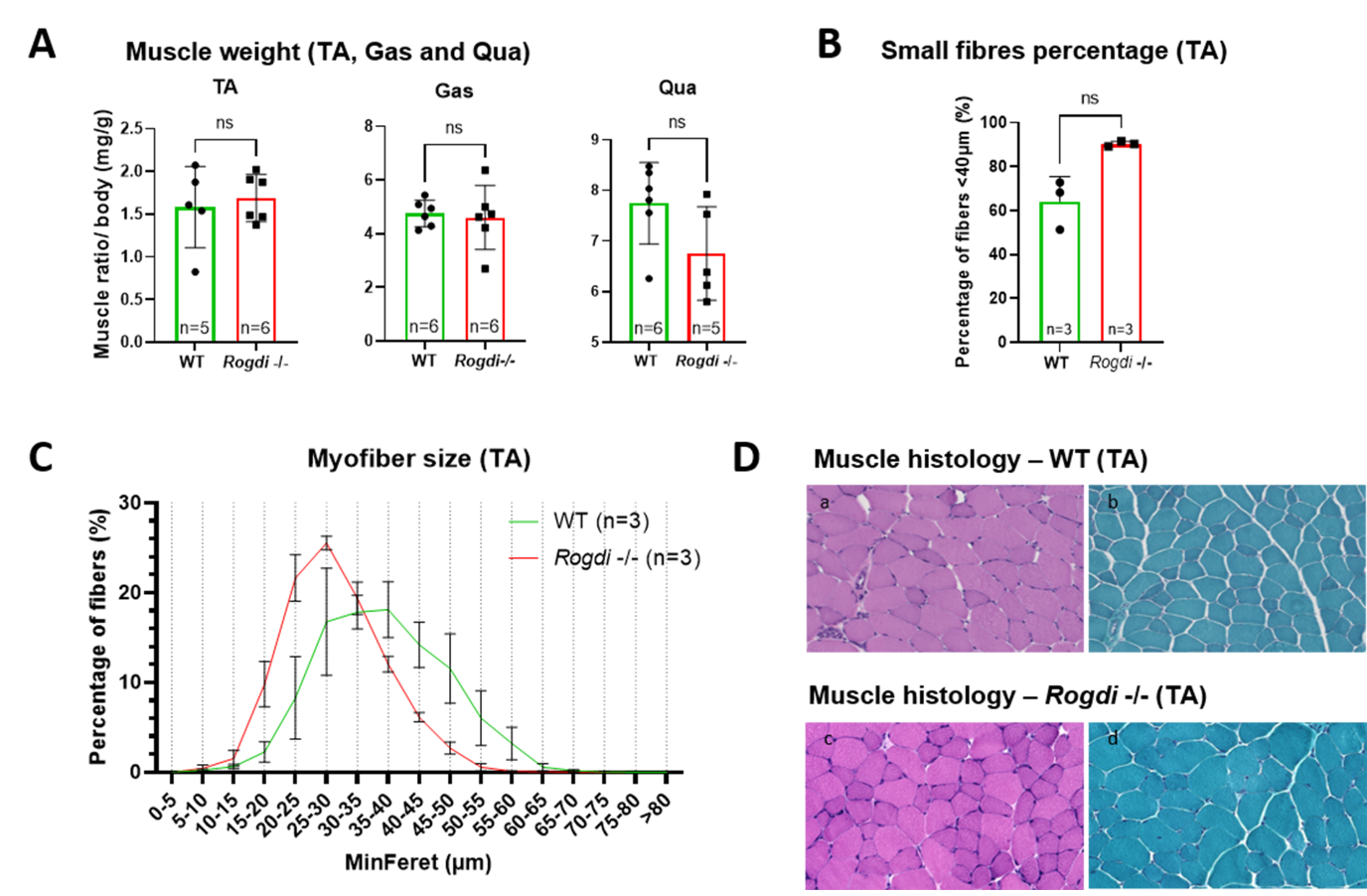
**

**Supplementary Figure S3. Muscle analysis.** Muscle/ body weight (A), percentage of small muscle fibers (B) and global MinFeret distributions (C) within control and *Rogdi ^-/-^* mice showed no difference at 8 weeks of age. Results are expressed as mean values ± SD. No difference in term of muscle histology was observed between a control and a *Rogdi ^-/-^* mouse (D) at 8 weeks of age [a: hematoxilin and eosin staining; b: gomori trichrome staining]. ns: not significant. TA: tibialis anterior; Gas: gastrocnemiens; Qua: quadriceps.


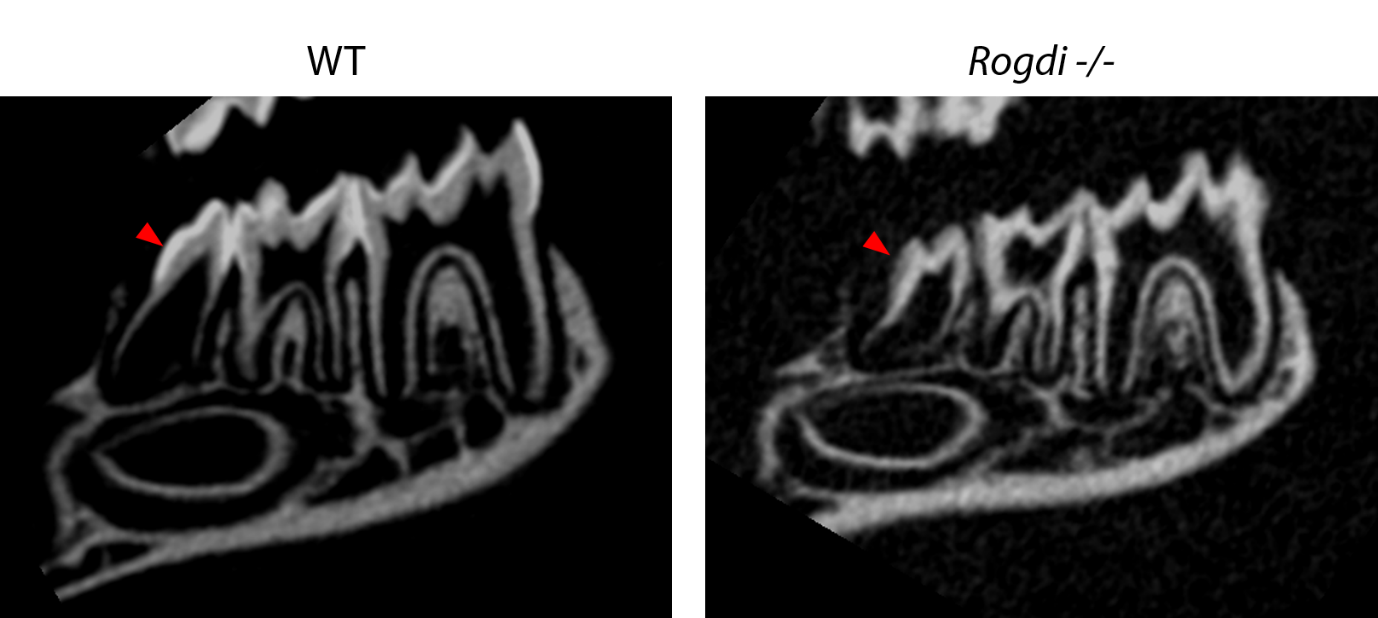


**Supplementary Figure S4. Micro-CT imaging of 4-week-old of WT and *Rogdi^-/-^* molars.** Optical sections in a sagittal plane show reduced enamel mineral density in the third molar of *Rogdi^-/-^* mice (red arrowheads).


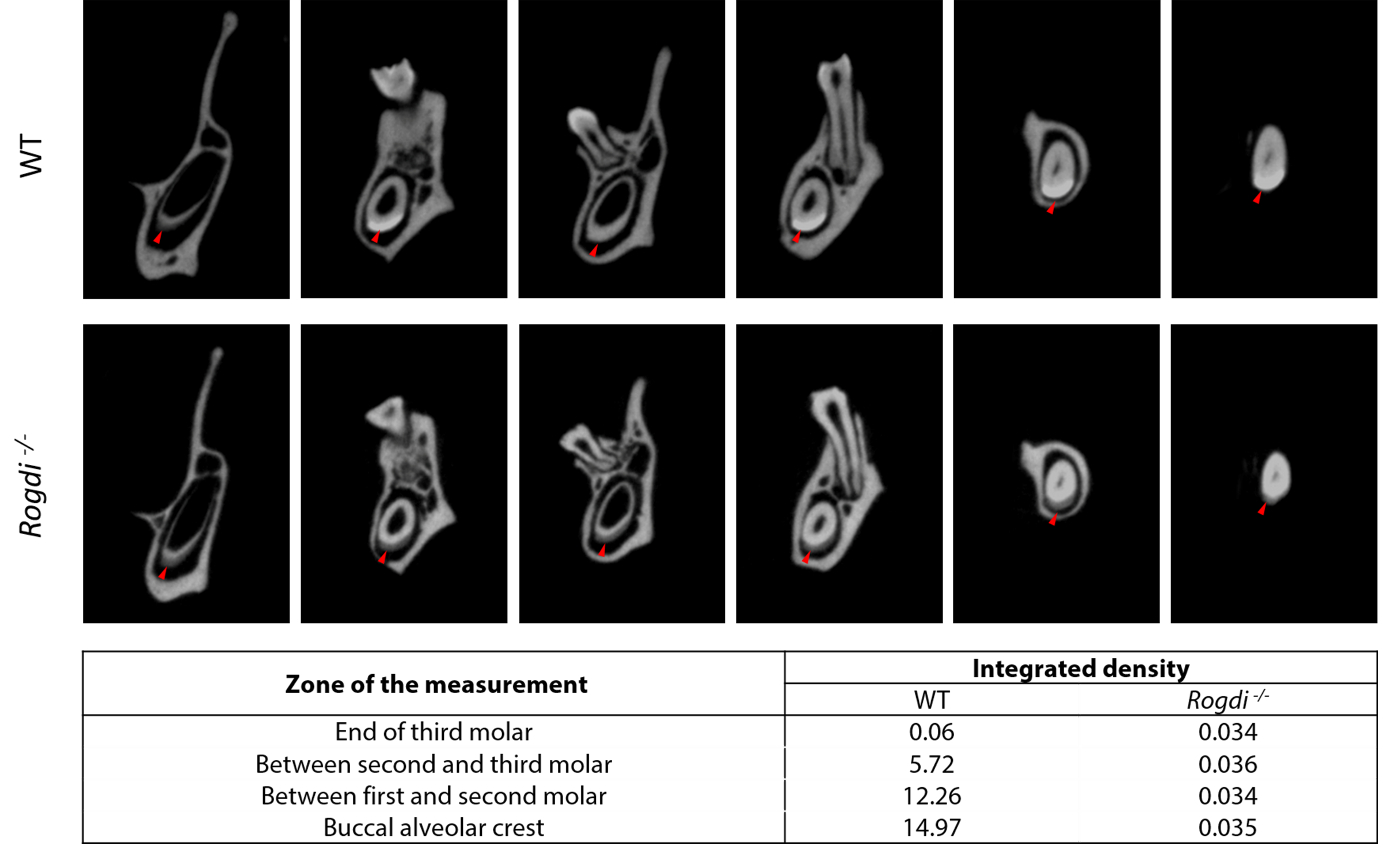


**Supplementary Figure S5. Micro-CT imaging of 8-week-old of WT and *Rogdi^-/-^* lower incisors.** Mandibular incisor cross sections show reduced enamel mineral density throughout amelogenesis. Table indicates the integrated density (the product of Area and Mean Gray Value) of WT and *Rogdi^-/-^* at different zones.


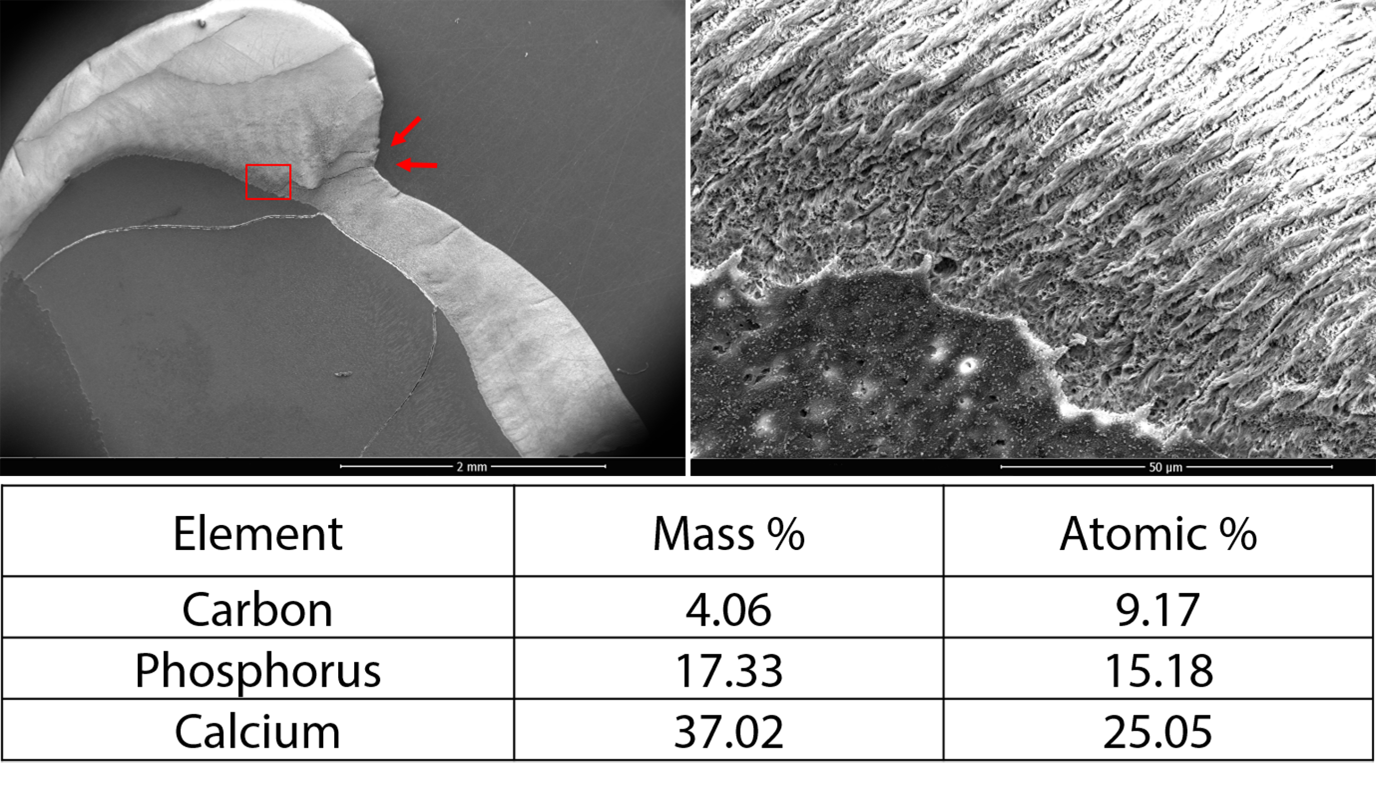


**Supplementary Figure S6. Scanning electron microscopy (SEM) of *ROGDI*-associated KTS patient Premolar (45).** Enamel was present, but this was hypoplastic (red arrows). Red boxed region show region in which SEM image was obtained. The enamel presents a clear decussating prism pattern. Table of energy dispersive X-ray spectrometry data for quantification of element display levels of element content in enamel. Calcium-phosphate ratio (Ca/P) was of 1.65.


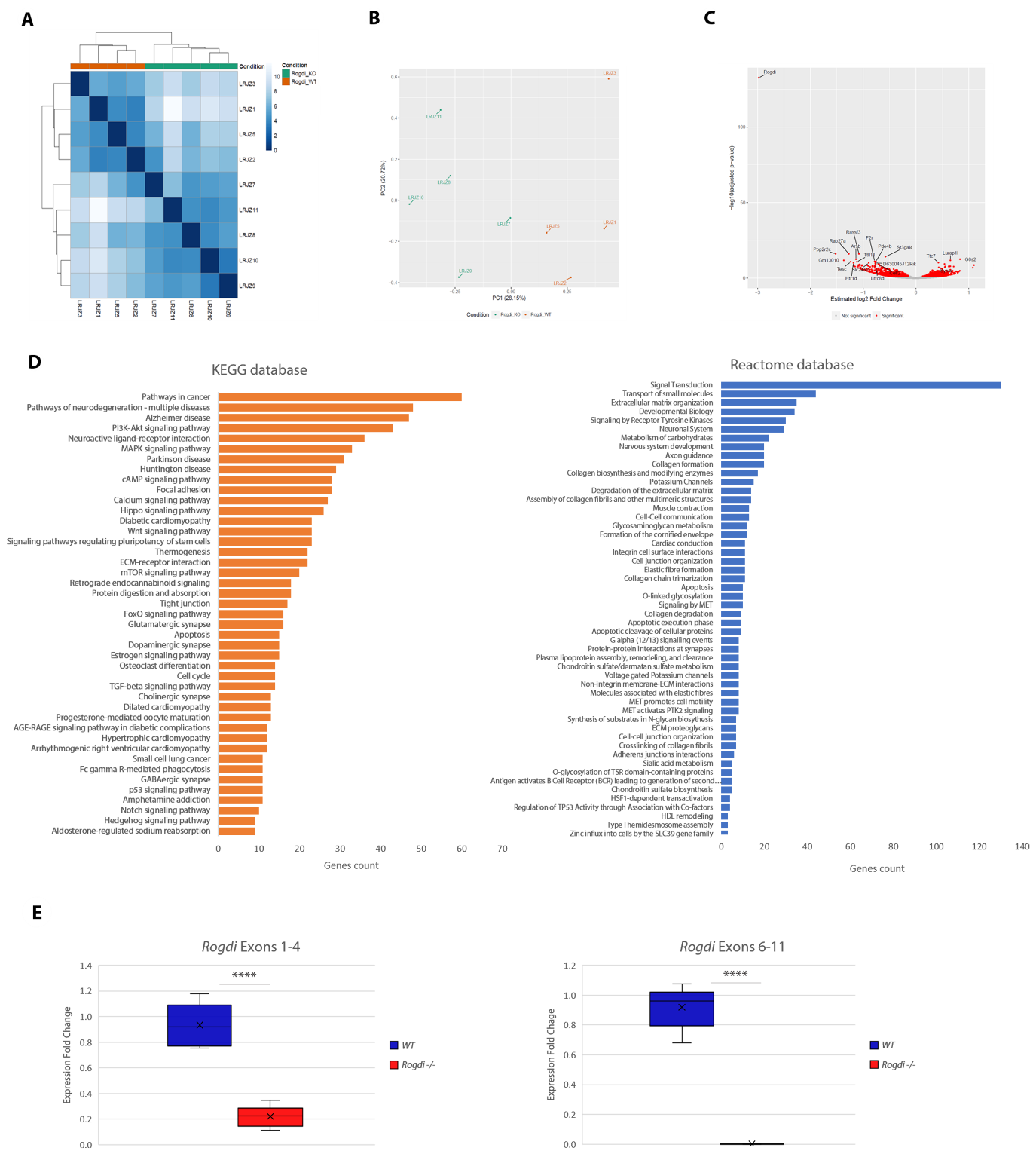


**Supplementary Figure S7. *Rogdi KO* vs WT comparison.** (A) Heatmap of sample-to-sample distances. Values correspond to the SERE ^1^ coefficient. The distances were computed from the raw counts. Hierarchical clustering was performed using the Unweighted Pair Group Method with Arithmetic mean (UPGMA) algorithm. (B) Principal component analysis. PCi axis represents the principal component i and the number into brackets indicates the percentage of explained variance associated with this axis. Principal Component Analysis was computed on variance stabilizing transformed data calculated with the method proposed in ^2^. (C) *Rogdi KO* vs WT volcano-plot representing -log10 (adjusted p-value) as a function of the estimated log2 Fold-Change. Significant genes were selected using the following threshold: adjusted p-value lower than 0.05. For significant genes, a selection of first gene names according to the adjusted p-value is displayed. (D) Kyoto Encyclopedia of Genes and Genomes (KEGG) and Reactome pathway enrichment analysis for up- and down-regulated genes between control vs *Rogdi^-/-^* samples. Analysis shows pathways of neurodegeneration, calcium signaling, synapse, transport of small molecules, and degradation of extracellular matrix, among others, were affected in *Rogdi^-/-^* mice. (E) RNA expression Fold change of *Rogdi*. RT-qPCR was performed on RNA extracted from postnatal 5-day lower incisors. Results were plotted on a graph with the expression of exons 1-4 (left) and 6-11 (right) of *Rogdi*. Fold-change was calculated based on mRNA relative expression of control group and normalized to *Gapdh* expression. ****p<0.0001.


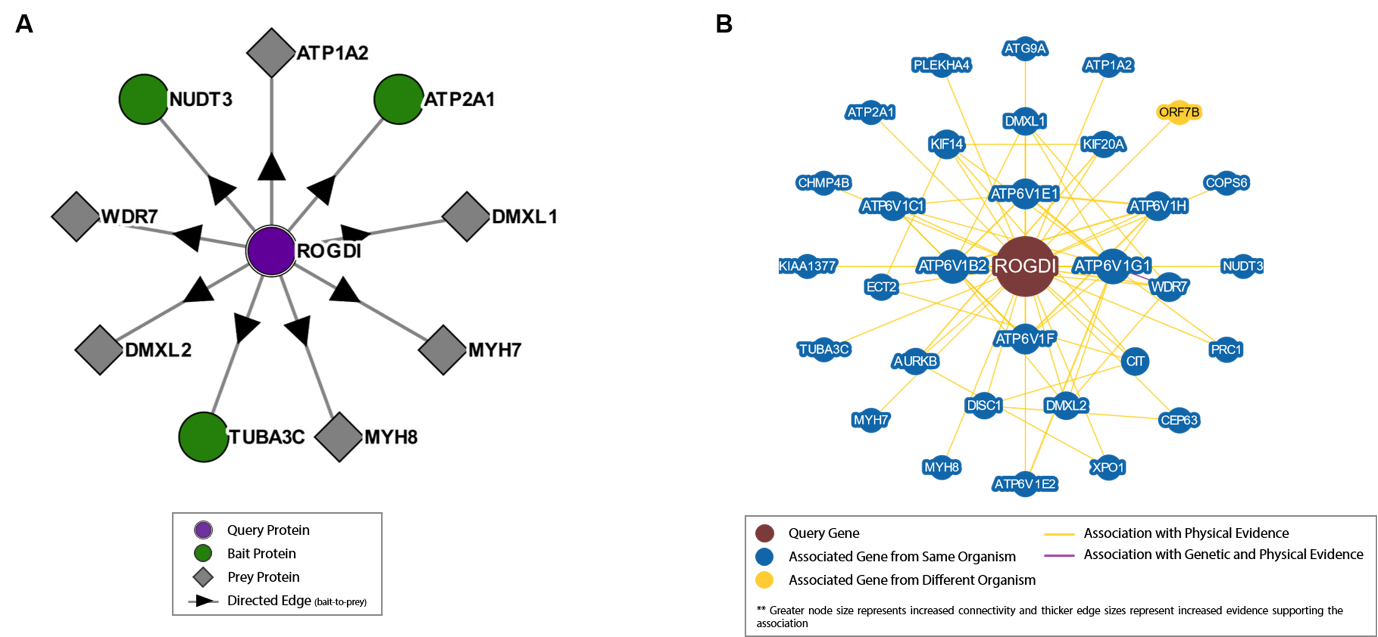


**Supplementary Figure S9. ROGDI protein interactions network.** (A) Rogdi interaction network in human cell lines HEK293T and HCT116 (Bioplex) ^3^. (B) Interaction protein network of ROGDI revealed by the BioGRID database (<https://thebiogrid.org>).

**Supplementary Table S1. *Rogdi* expression in 24 cell populations of the central nervous system** ^4^ **displayed from the highest expression to the lowest expression.**

| **Cell Type** | **Region** | ***Rogdi* expression (RPKM)** | **Cell function** |
| --- | --- | --- | --- |
| Corticostriatal Neurons | Cortex | 3168.8 | Motivated behavior ^5^ |
| Cholinergic Neurons | Basal Ganglion | 2388.9 | Regulate attention, memory, reward pathways, and motor activity ^6^ |
| Corticospinal, Corticopontine Neurons | Cortex | 2379.9 | Principal motor system for controlling movements that require the greatest skill and flexibility ^7^ |
| Neurons (CCK) | Cortex | 2065.9 | To regulate emotion and sculpt the network oscillations associated with cognition ^8^ |
| Corticothalamic Neurons | Cortex | 2022.0 | Involved in high-cognitive functions, such as decision-making and working memory ^9^ |
| Cholinergic Neurons | Forebrain | 1734.2 | Attentive functions ^10^ |
| Stellate & Basket Cells | Cerebellum | 1591.6 | Inhibitory GABAergic interneurons of the brain ^11^ |
| Motor Neurons | Brain Stem | 1583.1 | Alternative route to convey the information to the muscle targets in the periphery ^12^ |
| Drd1 Medium Spiny Neurons | Striatum | 1532.4 | GABAergic inhibitory cell ^13^ |
| Unipolar Brush Cell | Cerebellum | 1218.3 | Glutamatergic interneuron involved in the sensorimotor processes that regulate body, head and eye position, as well as in regions of the cochlear nucleus that process sensorimotor information ^14^ |
| Granule Cells | Cerebellum | 1030.9 | Involved in functions ranging from processing visual and motor information to learning and memory ^15^ |
| Interneurons (Cort) | Cortex | 993.6 | Ability to induce slow-wave sleep activity and regulated synaptic integration by augmenting the hyperpolarization-activated current IH ^16^ |
| Drd2 Medium Spiny Neurons | Striatum | 895.9 | GABAergic inhibitory cell ^13^ |
| Purkinje Cells | Cerebellum | 779.9 | Role in coordination, control, and learning of movements ^17^ |
| Interneurons (Pnoc) | Cortex | 697.7 | May be crucial for the maturation and neurogenesis of adult granule cells, as newly generated granule cells initially only receive GABAergic input from local interneurons ^18^ |
| Motor Neurons | Spinal Cord | 526.8 | Responsible for transmitting a movement-inducing signal from the upper motor neuron to the effector muscle ^12^ |
| Mature Oligodendrocytes & Progenitors | Cortex | 467.9 | Myelinating cells of the central nervous system (CNS) ^19^ |
| Golgi Cells | Cerebellum | 444.2 | Control on spatio-temporal signal organization and information storage in the granular layer playing a critical role for cerebellar computation ^20^ |
| Astrocytes | Cortex | 441.3 | Role in glutamate, ion (i.e., Ca^2+^, K^+^) and water homeostasis, defense against oxidative/nitrosative stress, energy storage, mitochondria biogenesis, scar formation, tissue repair via angiogenesis and neurogenesis, and synapse modulation ^21^ |
| Mature Oligodendrocytes | Cortex | 401.7 | Myelinating cells of the central nervous system (CNS) ^19^ |
| Mature Oligodendrocytes | Cerebellum | 327.9 | Myelinating cells of the central nervous system (CNS) ^19^ |
| Mature Oligodendrocytes & Progenitors | Cerebellum | 290.1 | Myelinating cells of the central nervous system (CNS) ^19^ |
| Bergmann Glia | Cerebellum | 185.3 | Role in controlling the membrane potential and thereby the activity of adjacent Purkinje cells ^22^ |
| Astrocytes | Cerebellum | 150.0 | Role in glutamate, ion (i.e., Ca^2+^, K^+^) and water homeostasis, defense against oxidative/nitrosative stress, energy storage, mitochondria biogenesis, scar formation, tissue repair via angiogenesis and neurogenesis, and synapse modulation ^21^ |

**Supplementary Table S2. Punnett square with WT, heterozygous and *Rogdi KO* ratio found and expected from heterozygous breeding.**

|  | **WT** | **KO** |
| --- | --- | --- |
| **WT** | **234 (24.7%; exp 25%)** | **517 (54.5%; exp 50%)** |
| **KO** | **517 (54.5%; exp 50%)** | **198 (20.9%; exp 25%)** |

**Supplementary Table S3. RNA sequencing.** Data are presented as log2 fold changes in *Rogdi^-/-^* vs. WT samples. For instance, a FC log2 value of −1.00 will correspond to a 50% reduction in mRNA level in the *Rogdi^-/-^* samples. Genes encoding regulators of tooth development, cell-cell adhesion and cell-matrix adhesion, proteins involved in ion transport such as calcium and transmembrane transport, nervous system development including hippocampus, synapse organization, synaptic transmission, learning, memory and locomotor behavior are often dysregulated in *Rogdi^-/-^* PN5 lower incisors.

| **Gene name** | | **Description** | | **Log2 FC**  **(*Rogdi^-/-^* vs WT)** | | ***p*-value** |
| --- | --- | --- | --- | --- | --- | --- |
| **Tooth development** | | | | | | |
| *Tspear* | thrombospondin type laminin G domain and EAR repeats | | -0.55 | | 4.4E-04 | |
| *Trp63* | transformation related protein 63 | | -0.53 | | 8.6E-06 | |
| *Nkx2-3* | NK2 homeobox 3 | | -0.50 | | 1.8E-03 | |
| *Edar* | ectodysplasin-A receptor | | -0.43 | | 1.7E-02 | |
| *Perp* | PERP. TP53 apoptosis effector | | -0.41 | | 1.0E-06 | |
| *Shh* | sonic hedgehog | | -0.37 | | 3.2E-02 | |
| *Apc* | APC. WNT signaling pathway regulator | | -0.35 | | 1.7E-05 | |
| *Acvr2a* | activin receptor IIA | | -0.31 | | 1.1E-03 | |
| *Wnt10a* | wingless-type MMTV integration site family. member 10A | | 0.32 | | 5.8E-03 | |
| *Foxc1* | forkhead box C1 | | 0.34 | | 2.1E-02 | |
| *Dll1* | delta like canonical Notch ligand 1 | | 0.36 | | 1.4E-02 | |
| *Twist1* | twist basic helix-loop-helix transcription factor 1 | | 0.38 | | 1.7E-03 | |
| *Phex* | phosphate regulating endopeptidase homolog. X-linked | | 0.38 | | 6.2E-05 | |
| *Dspp* | dentin sialophosphoprotein | | 0.39 | | 2.1E-04 | |
| *Tgfb1* | transforming growth factor. beta 1 | | 0.44 | | 1.6E-04 | |
| *Gas1* | growth arrest specific 1 | | 0.44 | | 7.6E-04 | |
| *Wnt6* | wingless-type MMTV integration site family. member 6 | | 0.44 | | 3.8E-04 | |
| *Hand2* | heart and neural crest derivatives expressed 2 | | 0.53 | | 1.2E-06 | |
| *Osr1* | odd-skipped related transcription factor 1 | | 0.54 | | 2.2E-03 | |
| *Id3* | inhibitor of DNA binding 3 | | 0.56 | | 1.8E-05 | |
| *Msx1* | msh homeobox 1 | | 0.56 | | 1.5E-07 | |
| *Col1a1* | collagen. type I. alpha 1 | | 0.62 | | 5.3E-11 | |
| **Cell-cell adhesion** | | | | | | |
| *Ctnna2* | catenin (cadherin associated protein). alpha 2 | | -1.18 | | 5.8E-09 | |
| *Nfasc* | neurofascin | | -1.08 | | 2.2E-07 | |
| *Lama3* | laminin. alpha 3 | | -1.01 | | 4.2E-09 | |
| *Kirrel2* | kirre like nephrin family adhesion molecule 2 | | -0.71 | | 2.0E-03 | |
| *Rap1gap* | Rap1 GTPase-activating protein | | -0.66 | | 6.6E-08 | |
| *Cdh1* | cadherin 1 | | -0.64 | | 1.3E-05 | |
| *Nectin4* | nectin cell adhesion molecule 4 | | -0.59 | | 3.9E-04 | |
| *Celsr2* | cadherin. EGF LAG seven-pass G-type receptor 2 | | -0.57 | | 2.7E-04 | |
| *Dsg1b* | desmoglein 1 beta | | -0.52 | | 4.1E-02 | |
| *Celsr1* | cadherin. EGF LAG seven-pass G-type receptor 1 | | -0.51 | | 5.0E-04 | |
| *Itga3* | integrin alpha 3 | | -0.50 | | 3.6E-04 | |
| *Pkp1* | plakophilin 1 | | -0.48 | | 7.4E-03 | |
| *Cdh6* | cadherin 6 | | -0.47 | | 4.3E-05 | |
| *Jag1* | jagged 1 | | -0.47 | | 1.4E-03 | |
| *Perp* | PERP. TP53 apoptosis effector | | -0.41 | | 8.9E-05 | |
| *Dsc3* | desmocollin 3 | | -0.41 | | 1.8E-03 | |
| *Cdh3* | cadherin 3 | | -0.38 | | 1.8E-02 | |
| *Prickle1* | prickle planar cell polarity protein 1 | | -0.36 | | 3.9E-06 | |
| *Abl2* | v-abl Abelson murine leukemia viral oncogene 2 (arg. Abelson-related gene) | | -0.34 | | 1.6E-04 | |
| *Srf* | serum response factor | | 0.30 | | 4.5E-02 | |
| *Cx3cl1* | chemokine (C-X3-C motif) ligand 1 | | 0.39 | | 8.9E-03 | |
| *Cldn5* | claudin 5 | | 0.40 | | 3.7E-02 | |
| *Tgfb1* | transforming growth factor. beta 1 | | 0.44 | | 4.8E-03 | |
| *Lims2* | LIM and senescent cell antigen like domains 2 | | 0.46 | | 3.7E-02 | |
| *Col13a1* | collagen. type XIII. alpha 1 | | 0.53 | | 1.9E-02 | |
| **Cell-matrix adhesion** | | | | | | |
| *Itgb6* | integrin beta 6 | | -0.89 | | 9.9E-07 | |
| *Fermt1* | fermitin family member 1 | | -0.58 | | 5.2E-03 | |
| *Itga3* | integrin alpha 3 | | -0.50 | | 3.6E-04 | |
| *Sirpa* | signal-regulatory protein alpha | | -0.42 | | 3.1E-04 | |
| *Npnt* | nephronectin | | -0.42 | | 7.1E-05 | |
| *Emp2* | epithelial membrane protein 2 | | -0.41 | | 9.3E-03 | |
| *Itgb4* | integrin beta 4 | | -0.33 | | 1.5E-02 | |
| *Srf* | serum response factor | | 0.30 | | 4.5E-02 | |
| *Col5a3* | collagen. type V. alpha 3 | | 0.40 | | 4.9E-02 | |
| *Emilin1* | elastin microfibril interfacer 1 | | 0.47 | | 1.3E-03 | |
| *Col13a1* | collagen. type XIII. alpha 1 | | 0.53 | | 1.9E-02 | |
| *Bcan* | brevican | | 0.63 | | 1.0E-08 | |
| **Hemidesmosome assembly** | | | | | | |
| *Lama3* | laminin. alpha 3 | | -1.01 | | 4.2E-09 | |
| *Col17a1* | collagen. type XVII. alpha 1 | | -0.36 | | 4.1E-04 | |
| *Itgb4* | integrin beta 4 | | -0.33 | | 1.5E-02 | |
| *Plec* | plectin | | 0.33 | | 3.3E-02 | |
| **Ion transport** | | | | | | |
| *Slc38a3* | solute carrier family 38. member 3 | | -1.09 | | 2.5E-09 | |
| *Slc39a4* | solute carrier family 39 (zinc transporter). member 4 | | -1.05 | | 1.1E-07 | |
| *Slc24a2* | solute carrier family 24 (sodium/potassium/calcium exchanger). member 2 | | -0.89 | | 8.7E-11 | |
| *Otop2* | otopetrin 2 | | -0.88 | | 6.4E-05 | |
| *Kcnh3* | potassium voltage-gated channel. subfamily H (eag-related). member 3 | | -0.86 | | 5.7E-05 | |
| *Slco4a1* | solute carrier organic anion transporter family. member 4a1 | | -0.85 | | 5.9E-05 | |
| *Lrrc8d* | leucine rich repeat containing 8D | | -0.77 | | 2.8E-10 | |
| *Steap1* | six transmembrane epithelial antigen of the prostate 1 | | -0.69 | | 3.4E-04 | |
| *Cacna2d2* | calcium channel. voltage-dependent. alpha 2/delta subunit 2 | | -0.65 | | 5.7E-03 | |
| *Slc24a3* | solute carrier family 24 (sodium/potassium/calcium exchanger). member 3 | | -0.63 | | 1.1E-03 | |
| *Fxyd3* | FXYD domain-containing ion transport regulator 3 | | -0.58 | | 6.3E-07 | |
| *Kcnip2* | Kv channel-interacting protein 2 | | -0.58 | | 1.1E-02 | |
| *Kcnk3* | potassium channel. subfamily K. member 3 | | -0.57 | | 6.5E-03 | |
| *Cldn17* | claudin 17 | | -0.56 | | 2.2E-02 | |
| *Kcnb2* | potassium voltage gated channel. Shab-related subfamily. member 2 | | -0.55 | | 5.4E-03 | |
| *Grik3* | glutamate receptor. ionotropic. kainate 3 | | -0.54 | | 2.4E-02 | |
| *Fxyd4* | FXYD domain-containing ion transport regulator 4 | | -0.53 | | 2.2E-03 | |
| *Itpr1* | inositol 1.4.5-trisphosphate receptor 1 | | -0.53 | | 2.6E-03 | |
| *Tmc7* | transmembrane channel-like gene family 7 | | -0.50 | | 2.8E-02 | |
| *Slc39a8* | solute carrier family 39 (metal ion transporter). member 8 | | -0.50 | | 6.8E-03 | |
| *Scnn1b* | sodium channel. nonvoltage-gated 1 beta | | -0.48 | | 3.2E-02 | |
| *Kcna3* | potassium voltage-gated channel. shaker-related subfamily. member 3 | | -0.47 | | 4.9E-02 | |
| *Slc41a3* | solute carrier family 41. member 3 | | -0.45 | | 1.7E-03 | |
| *Nipa2* | non-imprinted in Prader-Willi/Angelman syndrome 2 homolog (human) | | -0.45 | | 8.0E-03 | |
| *Kcnk1* | potassium channel. subfamily K. member 1 | | -0.43 | | 2.5E-04 | |
| *Nalcn* | sodium leak channel. non-selective | | -0.37 | | 3.8E-02 | |
| *Tmc4* | transmembrane channel-like gene family 4 | | -0.34 | | 2.4E-02 | |
| *Tmem38a* | transmembrane protein 38A | | -0.33 | | 4.7E-03 | |
| *Kcnh1* | potassium voltage-gated channel. subfamily H (eag-related). member 1 | | -0.32 | | 4.5E-02 | |
| *Lrrc8c* | leucine rich repeat containing 8 family. member C | | -0.31 | | 1.2E-03 | |
| *Slc39a11* | solute carrier family 39 (metal ion transporter). member 11 | | -0.31 | | 3.4E-04 | |
| *Slc12a4* | solute carrier family 12. member 4 | | -0.31 | | 9.5E-04 | |
| *Cacna1d* | calcium channel. voltage-dependent. L type. alpha 1D subunit | | -0.30 | | 2.6E-02 | |
| *Cnnm4* | cyclin M4 | | 0.30 | | 4.9E-02 | |
| *Calhm2* | calcium homeostasis modulator family member 2 | | 0.31 | | 4.6E-02 | |
| *Kcnn1* | potassium intermediate/small conductance calcium-activated channel. subfamily N. member 1 | | 0.34 | | 3.7E-02 | |
| *Kcnq4* | potassium voltage-gated channel. subfamily Q. member 4 | | 0.35 | | 3.2E-02 | |
| *Kcnc3* | potassium voltage gated channel. Shaw-related subfamily. member 3 | | 0.35 | | 4.5E-02 | |
| *Glrb* | glycine receptor. beta subunit | | 0.37 | | 1.8E-02 | |
| *Grin2d* | glutamate receptor. ionotropic. NMDA2D (epsilon 4) | | 0.39 | | 2.8E-02 | |
| *Best1* | bestrophin 1 | | 0.40 | | 4.2E-02 | |
| *Otop1* | otopetrin 1 | | 0.40 | | 4.4E-02 | |
| *Slc39a7* | solute carrier family 39 (zinc transporter). member 7 | | 0.41 | | 3.3E-02 | |
| *Kcnk2* | potassium channel. subfamily K. member 2 | | 0.44 | | 6.5E-04 | |
| *Scn1a* | sodium channel. voltage-gated. type I. alpha | | 0.46 | | 4.3E-02 | |
| *Slc38a11* | solute carrier family 38. member 11 | | 0.47 | | 4.9E-04 | |
| *Scn1b* | sodium channel. voltage-gated. type I. beta | | 0.47 | | 7.7E-03 | |
| *Orai1* | ORAI calcium release-activated calcium modulator 1 | | 0.47 | | 1.8E-03 | |
| *Kcnh2* | potassium voltage-gated channel. subfamily H (eag-related). member 2 | | 0.47 | | 2.5E-03 | |
| *Atp7b* | ATPase. Cu++ transporting. beta polypeptide | | 0.49 | | 3.7E-02 | |
| *Kcnj4* | potassium inwardly-rectifying channel. subfamily J. member 4 | | 0.51 | | 2.4E-02 | |
| *Kcnn4* | potassium intermediate/small conductance calcium-activated channel. subfamily N. member 4 | | 0.56 | | 1.1E-03 | |
| *Atp6v0c* | ATPase. H+ transporting. lysosomal V0 subunit C | | 0.60 | | 3.4E-04 | |
| **Calcium ion transport** | | | | | | |
| *Slc24a2* | solute carrier family 24 (sodium/potassium/calcium exchanger). member 2 | | -0.89 | | 8.7E-11 | |
| *F2r* | coagulation factor II (thrombin) receptor | | -0.78 | | 8.1E-12 | |
| *Cacna2d2* | calcium channel. voltage-dependent. alpha 2/delta subunit 2 | | -0.65 | | 5.7E-03 | |
| *Slc24a3* | solute carrier family 24 (sodium/potassium/calcium exchanger). member 3 | | -0.63 | | 1.1E-03 | |
| *Oprd1* | opioid receptor. delta 1 | | -0.54 | | 9.2E-03 | |
| *Wnk3* | WNK lysine deficient protein kinase 3 | | -0.54 | | 1.4E-03 | |
| *Itpr1* | inositol 1.4.5-trisphosphate receptor 1 | | -0.53 | | 2.6E-03 | |
| *Nalcn* | sodium leak channel. non-selective | | -0.37 | | 3.8E-02 | |
| *Cacna1d* | calcium channel. voltage-dependent. L type. alpha 1D subunit | | -0.30 | | 2.6E-02 | |
| *Rgs4* | regulator of G-protein signaling 4 | | 0.35 | | 3.7E-03 | |
| *Nos3* | nitric oxide synthase 3. endothelial cell | | 0.35 | | 2.1E-02 | |
| *Wfs1* | wolframin ER transmembrane glycoprotein | | 0.38 | | 3.7E-04 | |
| *Jak3* | Janus kinase 3 | | 0.39 | | 4.6E-02 | |
| *Best1* | bestrophin 1 | | 0.40 | | 4.2E-02 | |
| *Orai1* | ORAI calcium release-activated calcium modulator 1 | | 0.47 | | 1.8E-03 | |
| *Cdh23* | cadherin 23 (otocadherin) | | 0.50 | | 5.5E-05 | |
| *Kcnn4* | potassium intermediate/small conductance calcium-activated channel. subfamily N. member 4 | | 0.56 | | 1.1E-03 | |
| **Transmembrane transport** | | | | | | |
| *Slc39a4* | solute carrier family 39 (zinc transporter). member 4 | | -1.05 | | 1.1E-07 | |
| *Slc24a2* | solute carrier family 24 (sodium/potassium/calcium exchanger). member 2 | | -0.89 | | 8.7E-11 | |
| *Slc4a9* | solute carrier family 4. sodium bicarbonate cotransporter. member 9 | | -0.87 | | 8.9E-05 | |
| *Kcnh3* | potassium voltage-gated channel. subfamily H (eag-related). member 3 | | -0.86 | | 5.7E-05 | |
| *Slco4a1* | solute carrier organic anion transporter family. member 4a1 | | -0.85 | | 5.9E-05 | |
| *Sv2b* | synaptic vesicle glycoprotein 2 b | | -0.67 | | 2.7E-03 | |
| *Abcg5* | ATP binding cassette subfamily G member 5 | | -0.67 | | 5.4E-05 | |
| *Slc24a3* | solute carrier family 24 (sodium/potassium/calcium exchanger). member 3 | | -0.63 | | 1.1E-03 | |
| *Kcnb2* | potassium voltage gated channel. Shab-related subfamily. member 2 | | -0.55 | | 5.4E-03 | |
| *Itpr1* | inositol 1.4.5-trisphosphate receptor 1 | | -0.53 | | 2.6E-03 | |
| *Slc39a8* | solute carrier family 39 (metal ion transporter). member 8 | | -0.50 | | 6.8E-03 | |
| *Ano9* | anoctamin 9 | | -0.49 | | 4.9E-02 | |
| *Slc25a13* | solute carrier family 25 (mitochondrial carrier. adenine nucleotide translocator). member 13 | | -0.47 | | 6.4E-03 | |
| *Kcna3* | potassium voltage-gated channel. shaker-related subfamily. member 3 | | -0.47 | | 4.9E-02 | |
| *Abca4* | ATP-binding cassette. sub-family A (ABC1). member 4 | | -0.40 | | 2.9E-02 | |
| *Slc7a2* | solute carrier family 7 (cationic amino acid transporter. y+ system). member 2 | | -0.37 | | 2.1E-02 | |
| *Nalcn* | sodium leak channel. non-selective | | -0.37 | | 3.8E-02 | |
| *Mfsd14a* | major facilitator superfamily domain containing 14A | | -0.36 | | 7.5E-03 | |
| *Slc7a5* | solute carrier family 7 (cationic amino acid transporter. y+ system). member 5 | | -0.34 | | 3.5E-04 | |
| *Kcnh1* | potassium voltage-gated channel. subfamily H (eag-related). member 1 | | -0.32 | | 4.5E-02 | |
| *Slc39a11* | solute carrier family 39 (metal ion transporter). member 11 | | -0.31 | | 3.4E-04 | |
| *Slc12a4* | solute carrier family 12. member 4 | | -0.31 | | 9.5E-04 | |
| *Cacna1d* | calcium channel. voltage-dependent. L type. alpha 1D subunit | | -0.30 | | 2.6E-02 | |
| *Slc2a10* | solute carrier family 2 (facilitated glucose transporter). member 10 | | 0.30 | | 2.3E-02 | |
| *Slc25a19* | solute carrier family 25 (mitochondrial thiamine pyrophosphate carrier). member 19 | | 0.32 | | 4.2E-02 | |
| *Spns1* | spinster homolog 1 | | 0.32 | | 1.9E-02 | |
| *Abcg1* | ATP binding cassette subfamily G member 1 | | 0.33 | | 1.5E-02 | |
| *Kcnq4* | potassium voltage-gated channel. subfamily Q. member 4 | | 0.35 | | 3.2E-02 | |
| *Kcnc3* | potassium voltage gated channel. Shaw-related subfamily. member 3 | | 0.35 | | 4.5E-02 | |
| *Slc39a7* | solute carrier family 39 (zinc transporter). member 7 | | 0.41 | | 3.3E-02 | |
| *Scn1a* | sodium channel. voltage-gated. type I. alpha | | 0.46 | | 4.3E-02 | |
| *Kcnh2* | potassium voltage-gated channel. subfamily H (eag-related). member 2 | | 0.47 | | 2.5E-03 | |
| *Ano8* | anoctamin 8 | | 0.54 | | 1.9E-04 | |
| **Nervous system development** | | | | | | |
| *Plxna4* | | plexin A4 | | -1.05 | | 7.6E-07 |
| *Gpm6a* | | glycoprotein m6a | | -0.93 | | 3.0E-09 |
| *Rassf10* | | Ras association (RalGDS/AF-6) domain family (N-terminal) member 10 | | -0.70 | | 1.0E-05 |
| *Tox* | | thymocyte selection-associated high mobility group box | | -0.55 | | 8.6E-05 |
| *Alk* | | anaplastic lymphoma kinase | | -0.54 | | 3.1E-02 |
| *Igf2bp3* | | insulin-like growth factor 2 mRNA binding protein 3 | | -0.46 | | 1.0E-04 |
| *Nav2* | | neuron navigator 2 | | -0.41 | | 4.9E-02 |
| *Rtn4ip1* | | reticulon 4 interacting protein 1 | | -0.39 | | 2.0E-02 |
| *Ntrk2* | | neurotrophic tyrosine kinase. receptor. type 2 | | -0.37 | | 2.7E-02 |
| *Fzd6* | | frizzled class receptor 6 | | -0.36 | | 1.4E-02 |
| *Apc* | | APC. WNT signaling pathway regulator | | -0.35 | | 8.0E-04 |
| *Lmtk2* | | lemur tyrosine kinase 2 | | -0.34 | | 2.9E-02 |
| *Ophn1* | | oligophrenin 1 | | -0.33 | | 3.5E-03 |
| *Slit3* | | slit guidance ligand 3 | | 0.30 | | 3.1E-02 |
| *Dab1* | | disabled 1 | | 0.32 | | 9.4E-04 |
| *Zbtb45* | | zinc finger and BTB domain containing 45 | | 0.32 | | 3.1E-02 |
| *Insc* | | INSC spindle orientation adaptor protein | | 0.32 | | 4.9E-03 |
| *Bicdl1* | | BICD family like cargo adaptor 1 | | 0.32 | | 4.4E-02 |
| *Tfap2a* | | transcription factor AP-2. alpha | | 0.33 | | 2.3E-02 |
| *Sema7a* | | sema domain. immunoglobulin domain (Ig). and GPI membrane anchor. (semaphorin) 7A | | 0.35 | | 4.4E-04 |
| *Numbl* | | numb-like | | 0.36 | | 1.9E-02 |
| *Glrb* | | glycine receptor. beta subunit | | 0.37 | | 1.8E-02 |
| *Metrn* | | meteorin. glial cell differentiation regulator | | 0.39 | | 4.2E-02 |
| *Chac1* | | ChaC. cation transport regulator 1 | | 0.48 | | 7.2E-03 |
| *Sh2b2* | | SH2B adaptor protein 2 | | 0.48 | | 6.5E-03 |
| *Atoh8* | | atonal bHLH transcription factor 8 | | 0.51 | | 1.6E-03 |
| *Sema6b* | | sema domain. transmembrane domain (TM). and cytoplasmic domain. (semaphorin) 6B | | 0.57 | | 3.2E-04 |
| *Cer1* | | cerberus 1. DAN family BMP antagonist | | 0.57 | | 9.7E-03 |
| **Synapse organization** | | | | | | |
| *Nfasc* | | neurofascin | | -1.08 | | 2.2E-07 |
| *Gpm6a* | | glycoprotein m6a | | -0.93 | | 3.0E-09 |
| *Wnt3a* | | wingless-type MMTV integration site family. member 3A | | -0.71 | | 2.3E-03 |
| *Rims3* | | regulating synaptic membrane exocytosis 3 | | -0.64 | | 3.9E-04 |
| *Wnt7a* | | wingless-type MMTV integration site family. member 7A | | -0.59 | | 8.8E-04 |
| *Klk8* | | kallikrein related-peptidase 8 | | -0.54 | | 1.5E-02 |
| *Klk8* | | kallikrein related-peptidase 8 | | -0.54 | | 1.5E-02 |
| *Tuba1a* | | tubulin. alpha 1A | | -0.30 | | 4.6E-05 |
| *Tuba1a* | | tubulin. alpha 1A | | -0.30 | | 4.6E-05 |
| *Ptprs* | | protein tyrosine phosphatase. receptor type. S | | 0.33 | | 1.1E-02 |
| *Chrd* | | chordin | | 0.39 | | 2.8E-02 |
| *Lamb2* | | laminin. beta 2 | | 0.39 | | 1.1E-02 |
| *Mapt* | | microtubule-associated protein tau | | 0.41 | | 2.2E-02 |
| *Dvl1* | | dishevelled segment polarity protein 1 | | 0.41 | | 4.6E-05 |
| *Shank3* | | SH3 and multiple ankyrin repeat domains 3 | | 0.45 | | 4.5E-03 |
| *C1ql3* | | C1q-like 3 | | 0.48 | | 5.9E-03 |
| *Shank2* | | SH3 and multiple ankyrin repeat domains 2 | | 0.49 | | 1.1E-05 |
| *Apoe* | | apolipoprotein E | | 0.59 | | 1.8E-04 |
| *Bsn* | | bassoon | | 0.61 | | 8.1E-03 |
| **Chemical synaptic transmission** | | | | | | |
| *Htr1d* | | 5-hydroxytryptamine (serotonin) receptor 1D | | -1.18 | | 8.5E-11 |
| *Cntnap2* | | contactin associated protein-like 2 | | -0.93 | | 9.9E-07 |
| *Sv2b* | | synaptic vesicle glycoprotein 2 b | | -0.67 | | 2.7E-03 |
| *Ston2* | | stonin 2 | | -0.65 | | 1.0E-04 |
| *Slc12a4* | | solute carrier family 12. member 4 | | -0.31 | | 9.5E-04 |
| *Glrb* | | glycine receptor. beta subunit | | 0.37 | | 1.8E-02 |
| *Htr1b* | | 5-hydroxytryptamine (serotonin) receptor 1B | | 0.59 | | 4.2E-03 |
| *Tpgs1* | | tubulin polyglutamylase complex subunit 1 | | 0.62 | | 9.1E-03 |
| **Glutamatergic synaptic transmission** | | | | | | |
| *Ntrk2* | | neurotrophic tyrosine kinase. receptor. type 2 | | -0.37 | | 2.7E-02 |
| *Grin2d* | | glutamate receptor. ionotropic. NMDA2D (epsilon 4) | | 0.39 | | 2.8E-02 |
| *Shank3* | | SH3 and multiple ankyrin repeat domains 3 | | 0.45 | | 4.5E-03 |
| *Shank2* | | SH3 and multiple ankyrin repeat domains 2 | | 0.49 | | 1.1E-05 |
| **Synaptic vesicle endocytosis** | | | | | | |
| *Cd24a* | | CD24a antigen | | -0.87 | | 5.5E-05 |
| *Sh3gl2* | | SH3-domain GRB2-like 2 | | -0.67 | | 1.5E-06 |
| *Ston2* | | stonin 2 | | -0.65 | | 1.0E-04 |
| *Amph* | | amphiphysin | | -0.46 | | 2.6E-03 |
| *Ophn1* | | oligophrenin 1 | | -0.33 | | 3.5E-03 |
| **Regulation of synaptic vesicle exocytosis** | | | | | | |
| *Htr1d* | | 5-hydroxytryptamine (serotonin) receptor 1D | | -1.18 | | 8.5E-11 |
| *Sv2b* | | synaptic vesicle glycoprotein 2 b | | -0.67 | | 2.7E-03 |
| *Htr1b* | | 5-hydroxytryptamine (serotonin) receptor 1B | | 0.59 | | 4.2E-03 |
| *Rims3* | | regulating synaptic membrane exocytosis 3 | | -0.64 | | 3.9E-04 |
| *Wnt7a* | | wingless-type MMTV integration site family. member 7A | | -0.59 | | 8.8E-04 |
| *Pfn2* | | profilin 2 | | -0.38 | | 3.8E-06 |
| *Kcnh1* | | potassium voltage-gated channel. subfamily H (eag-related). member 1 | | -0.32 | | 4.5E-02 |
| *Rab3a* | | RAB3A. member RAS oncogene family | | 0.38 | | 6.0E-03 |
| *Dvl1* | | dishevelled segment polarity protein 1 | | 0.41 | | 4.6E-05 |
| *Rims1* | | regulating synaptic membrane exocytosis 1 | | 0.69 | | 1.3E-05 |
| **Hippocampus development** | | | | | | |
| *Wnt3a* | | wingless-type MMTV integration site family. member 3A | | -0.71 | | 2.3E-03 |
| *Alk* | | anaplastic lymphoma kinase | | -0.54 | | 3.1E-02 |
| *Trp73* | | transformation related protein 73 | | -0.38 | | 1.9E-02 |
| *Tuba1a* | | tubulin. alpha 1A | | -0.30 | | 4.6E-05 |
| *Srf* | | serum response factor | | 0.30 | | 4.5E-02 |
| *Dab1* | | disabled 1 | | 0.32 | | 9.4E-04 |
| *Ptprs* | | protein tyrosine phosphatase. receptor type. S | | 0.33 | | 1.1E-02 |
| *Anxa3* | | annexin A3 | | 0.34 | | 1.1E-02 |
| *Uqcrq* | | ubiquinol-cytochrome c reductase. complex III subunit VII | | 0.51 | | 2.1E-02 |
| *Sema6b* | | sema domain. transmembrane domain (TM). and cytoplasmic domain. (semaphorin) 6B | | 0.57 | | 3.2E-04 |
| *Sct* | | secretin | | 0.61 | | 5.4E-04 |
| *Bcan* | | brevican | | 0.63 | | 1.0E-08 |
| **Learning or memory** | | | | | | |
| *Cntnap2* | | contactin associated protein-like 2 | | -0.93 | | 9.9E-07 |
| *Slc24a2* | | solute carrier family 24 (sodium/potassium/calcium exchanger). member 2 | | -0.89 | | 8.7E-11 |
| *Crhr1* | | corticotropin releasing hormone receptor 1 | | -0.89 | | 5.7E-05 |
| *Th* | | tyrosine hydroxylase | | -0.86 | | 3.0E-05 |
| *Pak6* | | p21 (RAC1) activated kinase 6 | | -0.84 | | 1.5E-06 |
| *Jph4* | | junctophilin 4 | | -0.83 | | 2.5E-08 |
| *Klk8* | | kallikrein related-peptidase 8 | | -0.54 | | 1.5E-02 |
| *Itga3* | | integrin alpha 3 | | -0.50 | | 3.6E-04 |
| *Amph* | | amphiphysin | | -0.46 | | 2.6E-03 |
| *Stra6* | | stimulated by retinoic acid gene 6 | | -0.40 | | 1.8E-02 |
| *Zfp385a* | | zinc finger protein 385A | | -0.38 | | 1.6E-02 |
| *Ntrk2* | | neurotrophic tyrosine kinase. receptor. type 2 | | -0.37 | | 2.7E-02 |
| *Nedd9* | | neural precursor cell expressed. developmentally down-regulated gene 9 | | -0.35 | | 5.3E-06 |
| *Abl2* | | v-abl Abelson murine leukemia viral oncogene 2 (arg. Abelson-related gene) | | -0.34 | | 1.6E-04 |
| *Prnp* | | prion protein | | -0.34 | | 2.4E-03 |
| *Tuba1a* | | tubulin. alpha 1A | | -0.30 | | 4.6E-05 |
| *Igf2* | | insulin-like growth factor 2 | | 0.35 | | 9.5E-03 |
| *Mapt* | | microtubule-associated protein tau | | 0.41 | | 2.2E-02 |
| *Kcnk2* | | potassium channel. subfamily K. member 2 | | 0.44 | | 6.5E-04 |
| *Ptprz1* | | protein tyrosine phosphatase. receptor type Z. polypeptide 1 | | 0.45 | | 1.6E-03 |
| *Shank3* | | SH3 and multiple ankyrin repeat domains 3 | | 0.45 | | 4.5E-03 |
| *Shank2* | | SH3 and multiple ankyrin repeat domains 2 | | 0.49 | | 1.1E-05 |
| *Foxo6* | | forkhead box O6 | | 0.53 | | 2.7E-02 |
| *Jun* | | jun proto-oncogene | | 0.63 | | 6.4E-05 |
| *Jph3* | | junctophilin 3 | | 0.65 | | 5.8E-03 |
| *Nog* | | noggin | | 0.76 | | 3.1E-05 |
| **Locomotory behavior** | | | | | | |
| *Cntnap2* | | contactin associated protein-like 2 | | -0.93 | | 9.9E-07 |
| *Th* | | tyrosine hydroxylase | | -0.86 | | 3.0E-05 |
| *Pak6* | | p21 (RAC1) activated kinase 6 | | -0.84 | | 1.5E-06 |
| *Slc6a3* | | solute carrier family 6 (neurotransmitter transporter. dopamine). member 3 | | -0.59 | | 1.3E-02 |
| *Celsr1* | | cadherin. EGF LAG seven-pass G-type receptor 1 | | -0.51 | | 5.0E-04 |
| *Nav2* | | neuron navigator 2 | | -0.41 | | 4.9E-02 |
| *Zfp385a* | | zinc finger protein 385A | | -0.38 | | 1.6E-02 |
| *Zdhhc8* | | zinc finger. DHHC domain containing 8 | | 0.30 | | 3.3E-03 |
| *Shank3* | | SH3 and multiple ankyrin repeat domains 3 | | 0.45 | | 4.5E-03 |
| *Cdh23* | | cadherin 23 (otocadherin) | | 0.50 | | 5.5E-05 |
| **Wnt signaling** | | | | | | |
| *Wnt3a* | | wingless-type MMTV integration site family. member 3A | | -0.71 | | 2.3E-03 |
| *Wnt7a* | | wingless-type MMTV integration site family. member 7A | | -0.59 | | 8.8E-04 |
| *Celsr2* | | cadherin. EGF LAG seven-pass G-type receptor 2 | | -0.57 | | 2.7E-04 |
| *Apcdd1* | | adenomatosis polyposis coli down-regulated 1 | | -0.48 | | 5.4E-04 |
| *Cxxc4* | | CXXC finger 4 | | -0.47 | | 2.3E-02 |
| *Tle4* | | transducin-like enhancer of split 4 | | -0.37 | | 3.2E-03 |
| *Prickle1* | | prickle planar cell polarity protein 1 | | -0.36 | | 3.9E-06 |
| *Fzd6* | | frizzled class receptor 6 | | -0.36 | | 1.4E-02 |
| *Apc* | | APC. WNT signaling pathway regulator | | -0.35 | | 8.0E-04 |
| *Myc* | | myelocytomatosis oncogene | | 0.32 | | 1.4E-02 |
| *Pias4* | | protein inhibitor of activated STAT 4 | | 0.32 | | 2.3E-02 |
| *Cpz* | | carboxypeptidase Z | | 0.35 | | 3.7E-02 |
| *Fzd8* | | frizzled class receptor 8 | | 0.39 | | 1.1E-02 |
| *Dvl1* | | dishevelled segment polarity protein 1 | | 0.41 | | 4.6E-05 |
| *Wnt6* | | wingless-type MMTV integration site family. member 6 | | 0.44 | | 8.7E-03 |
| *Lzts2* | | leucine zipper. putative tumor suppressor 2 | | 0.49 | | 4.0E-03 |
| *Hic1* | | hypermethylated in cancer 1 | | 0.50 | | 2.4E-02 |
| *Wnt10b* | | wingless-type MMTV integration site family. member 10B | | 0.50 | | 1.8E-02 |
| *Gsc* | | goosecoid homeobox | | 0.57 | | 1.8E-02 |
| **Notch signaling** | | | | | | |
| *Tspear* | | thrombospondin type laminin G domain and EAR repeats | | -0.55 | | 9.7E-03 |
| *Trp63* | | transformation related protein 63 | | -0.53 | | 4.6E-04 |
| *Jag1* | | jagged 1 | | -0.47 | | 1.4E-03 |
| *Cdh6* | | cadherin 6 | | -0.47 | | 4.3E-05 |
| *Perp* | | PERP. TP53 apoptosis effector | | -0.41 | | 8.9E-05 |
| *Arrb1* | | arrestin. beta 1 | | -0.39 | | 1.4E-02 |
| *Ptp4a3* | | protein tyrosine phosphatase 4a3 | | 0.32 | | 4.0E-02 |
| *Notch4* | | notch 4 | | 0.39 | | 1.4E-02 |
| *Zbtb7a* | | zinc finger and BTB domain containing 7a | | 0.39 | | 2.9E-02 |
| *Mib2* | | mindbomb E3 ubiquitin protein ligase 2 | | 0.39 | | 1.2E-02 |
| *Tcim* | | transcriptional and immune response regulator | | 0.43 | | 7.4E-08 |
| *Tgfb1* | | transforming growth factor. beta 1 | | 0.44 | | 4.8E-03 |
| *Akt1s1* | | AKT1 substrate 1 (proline-rich) | | 0.46 | | 3.3E-02 |
| *Chac1* | | ChaC. cation transport regulator 1 | | 0.48 | | 7.2E-03 |
| *Dtx1* | | deltex 1. E3 ubiquitin ligase | | 0.54 | | 8.8E-03 |
| *Slc16a12* | solute carrier family 16 (monocarboxylic acid transporters). member 12 | | 0.54 | | 6.4E-03 | |

**Supplementary Table S4. Primers sequence used for RT-qPCR**

| **Genes** | **Forward primer** | **Reverse primer** |
| --- | --- | --- |
| *Rogdi* exons 1-4 | 5’ - GGAGGAGGAGTTTCGCTGGT | 5’ - CCTGGGAGAGTGAAGCGGAG |
| *Rogdi* exons 6-11 | 5’ - GCAACCCACCTCCACCAAGA | 5’ - AGAGTTGCAGGGAGACGGTG |
| *Gapdh* | 5’ - CCACCCATGGCAAATTCCATGGCA | 5’ - TCTAGACGGCAGGTCAGGTCCACC |

**References**

1. Schulze, S. K., Kanwar, R., Gölzenleuchter, M., Therneau, T. M. & Beutler, A. S. SERE: Single-parameter quality control and sample comparison for RNA-Seq. *BMC Genomics* **13**, 524 (2012).

2. Love, M. I., Huber, W. & Anders, S. Moderated estimation of fold change and dispersion for RNA-seq data with DESeq2. *Genome Biology* **15**, 550 (2014).

3. Schweppe, D. K., Huttlin, E. L., Harper, J. W. & Gygi, S. P. BioPlex Display: An Interactive Suite for Large-Scale AP–MS Protein–Protein Interaction Data. *J. Proteome Res.* **17**, 722–726 (2018).

4. Doyle, J. P. *et al.* Application of a Translational Profiling Approach for the Comparative Analysis of CNS Cell Types. *Cell* **135**, 749–762 (2008).

5. Shepherd, G. M. G. Corticostriatal connectivity and its role in disease. *Nat Rev Neurosci* **14**, 278–291 (2013).

6. Allaway, K. C. & Machold, R. Developmental specification of forebrain cholinergic neurons. *Developmental Biology* **421**, 1–7 (2017).

7. Martin, J. H. The corticospinal system: from development to motor control. *Neuroscientist* **11**, 161–173 (2005).

8. Whissell, P. D. *et al.* Selective Activation of Cholecystokinin-Expressing GABA (CCK-GABA) Neurons Enhances Memory and Cognition. *eNeuro* **6**, (2019).

9. Vaasjo, L. O. *et al.* Characterization and manipulation of Corticothalamic neurons in associative cortices using Syt6-Cre transgenic mice. *J Comp Neurol* **530**, 1020–1048 (2022).

10. Villano, I. *et al.* Basal Forebrain Cholinergic System and Orexin Neurons: Effects on Attention. *Front Behav Neurosci* **11**, 10 (2017).

11. Li, W. *et al.* Dendritic Inhibition by Shh Signaling-Dependent Stellate Cell Pool Is Critical for Motor Learning. *J Neurosci* **42**, 5130–5143 (2022).

12. Stifani, N. Motor neurons and the generation of spinal motor neuron diversity. *Front Cell Neurosci* **8**, 293 (2014).

13. Gangarossa, G. *et al.* Distribution and compartmental organization of GABAergic medium-sized spiny neurons in the mouse nucleus accumbens. *Front Neural Circuits* **7**, 22 (2013).

14. Mugnaini, E., Sekerková, G. & Martina, M. The unipolar brush cell: a remarkable neuron finally receiving deserved attention. *Brain Res Rev* **66**, 220–245 (2011).

15. Giovannucci, A. *et al.* Cerebellar granule cells acquire a widespread predictive feedback signal during motor learning. *Nat Neurosci* **20**, 727–734 (2017).

16. Maynard, K. R. *et al.* TrkB Signaling Influences Gene Expression in Cortistatin-Expressing Interneurons. *eNeuro* **7**, ENEURO.0310-19.2019 (2020).

17. Kano, M. & Watanabe, M. Chapter 4 - Cerebellar circuits. in *Neural Circuit and Cognitive Development (Second Edition)* (eds. Rubenstein, J., Rakic, P., Chen, B. & Kwan, K. Y.) 79–102 (Academic Press, 2020). doi:10.1016/B978-0-12-814411-4.00004-4.

18. Miller, J. A. *et al.* Conserved molecular signatures of neurogenesis in the hippocampal subgranular zone of rodents and primates. *Development* **140**, 4633–4644 (2013).

19. Kuhn, S., Gritti, L., Crooks, D. & Dombrowski, Y. Oligodendrocytes in Development, Myelin Generation and Beyond. *Cells* **8**, 1424 (2019).

20. D’Angelo, E. The critical role of Golgi cells in regulating spatio-temporal integration and plasticity at the cerebellum input stage. *Front Neurosci* **2**, 35–46 (2008).

21. Kim, Y., Park, J. & Choi, Y. K. The Role of Astrocytes in the Central Nervous System Focused on BK Channel and Heme Oxygenase Metabolites: A Review. *Antioxidants (Basel)* **8**, 121 (2019).

22. Wang, F., Xu, Q., Wang, W., Takano, T. & Nedergaard, M. Bergmann glia modulate cerebellar Purkinje cell bistability via Ca2+-dependent K+ uptake. *Proc Natl Acad Sci U S A* **109**, 7911–7916 (2012).
